# Pathology of pain and its implications for therapeutic interventions

**DOI:** 10.1038/s41392-024-01845-w

**Published:** 2024-06-08

**Authors:** Bo Cao, Qixuan Xu, Yajiao Shi, Ruiyang Zhao, Hanghang Li, Jie Zheng, Fengyu Liu, You Wan, Bo Wei

**Affiliations:** 1 Department of General Surgery, First Medical Center, Chinese PLA General Hospital, Beijing, 100853 China; 2 Medical School of Chinese PLA, Beijing, 100853 China; 3 Neuroscience Research Institute and Department of Neurobiology, School of Basic Medical Sciences, Key Laboratory for Neuroscience, Ministry of Education/National Health Commission, Peking University, Beijing, 100191 China

**Keywords:** Diseases, Neuroscience

## Abstract

Pain is estimated to affect more than 20% of the global population, imposing incalculable health and economic burdens. Effective pain management is crucial for individuals suffering from pain. However, the current methods for pain assessment and treatment fall short of clinical needs. Benefiting from advances in neuroscience and biotechnology, the neuronal circuits and molecular mechanisms critically involved in pain modulation have been elucidated. These research achievements have incited progress in identifying new diagnostic and therapeutic targets. In this review, we first introduce fundamental knowledge about pain, setting the stage for the subsequent contents. The review next delves into the molecular mechanisms underlying pain disorders, including gene mutation, epigenetic modification, posttranslational modification, inflammasome, signaling pathways and microbiota. To better present a comprehensive view of pain research, two prominent issues, sexual dimorphism and pain comorbidities, are discussed in detail based on current findings. The status quo of pain evaluation and manipulation is summarized. A series of improved and innovative pain management strategies, such as gene therapy, monoclonal antibody, brain-computer interface and microbial intervention, are making strides towards clinical application. We highlight existing limitations and future directions for enhancing the quality of preclinical and clinical research. Efforts to decipher the complexities of pain pathology will be instrumental in translating scientific discoveries into clinical practice, thereby improving pain management from bench to bedside.

## Introduction

Pain is defined as an unpleasant sensory and emotional experience associated with, or resembling that associated with, actual or potential tissue damage.^1^ It is considered the most primitive and widespread human experience. Owing to its subjective nature, the interplay of nociceptive, cognitive, emotional and social components collectively shapes the pain experience.^2^ Acute pain acts as a defense mechanism against noxious stimuli, infection, homeostasis dysfunction and secondary insults.^3,4^ Patients suffering from congenital insensitivity lack the ability to avoid damage, potentially leading to a predisposition toward self-mutilation.^5^ In contrast, chronic pain is inherently distressing and often the primary reason for patients to seek medical care. It poses a vast socioeconomic burden globally,^6^ with prevalence rates ranging from 10% to 40% and a relatively low recovery rate of only 5%.^7–9^ Pain relief has been a requisite and an important index for clinical treatment.

Pain serves as a crucial nexus between primary diseases and secondary outcomes. It can trigger a dynamic and detrimental interplay among biological, social and psychological factors, leading to disability and poor prognosis for patients. Pain-related psychiatric disorders, such as insomnia, depression, anxiety and impaired social interaction, can exacerbate the progression of primary diseases. These pathological deteriorations also negatively impact social relationships and self-esteem as evidenced by notable increases in divorce, substance abuse, and suicide rates.^10–12^ Chronic pain also undermines the survival benefits of cancer treatment.^13^ Notably, pain is not equal to suffering. The outcomes of pain are affected by various factors unique to an individual. For instance, massage can elicit pleasant sensations despite transient pain, and an individual in a positive emotional state may exhibit greater pain tolerance. These examples underscore that pain extends beyond a mere biological event and is intricately processed by the brain.

Analgesic drugs are the mainstay of acute and chronic pain management. Despite their short-term effectiveness, significant concerns regarding drug dependence, addiction and other side effects have been raised.^14,15^ The misuse of analgesics has also garnered international attention. New insights into the mechanisms underlying pain sensitivity and recovery are gradually being reported. The development of new therapeutic modalities, drug delivery systems and nonpharmaceutical adjuvant therapies has potential value in pain management. However, these varied interventions still fall short of fully addressing the needs of an individual’s quality of life.

This review will introduce the basic knowledge concerning pain research and then discuss current advances in understanding the pathology of pain perception and modulation. Two hot topics, sexual dimorphism and pain comorbidity, will also be discussed. Management approaches for pain will be summarized and remarked for fully displaying the status quo of pain research. Finally, we will discuss the existing limitations and propose future directions for enhancing the research and clinical practice of pain.

## Historical milestones of investigations into pain therapy

**Fig. 1:**
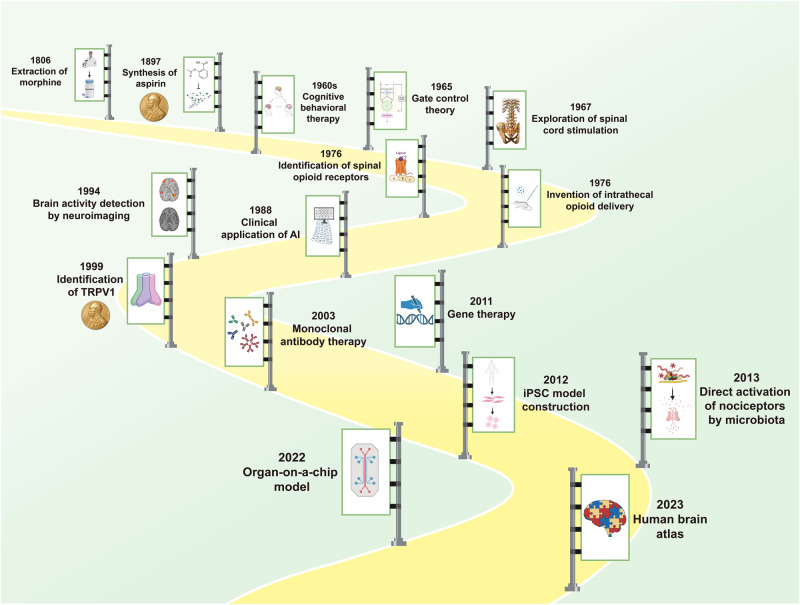
The brief timeline of historic milestones in the field of pain therapy. Morphine was first extracted in 1806, which opened the chapter in fighting with pain using the fruits of modern medicine. Since then, many intervention methods for pain management were discovered and came into clinical application, such as CBT, spinal cord stimulation, monoclonal antibody therapy and gene therapy. The progress in the research on pain mechanisms and interdisciplinary collaboration boosted advances in pain therapy. In recent years, the wide application of high-throughput biotechnologies has further deepened the understanding in pain pathology and has contributed to the development of individualized pain management. Key milestones of pain therapy are chronologically illustrated in the figure. The achievements awarded by the Nobel prizes are marked with the medals

The history of human development is intertwined with the struggles against pain (Fig. 1). Opioid alkaloids, derived from the opium poppy, have been used for analgesia and euphoria for thousands of years. In 1806, Friedrich W. Sertürner pioneered the extraction of pure opioids. This event opened a new chapter in fighting with pain using modern medicine. Another representative drug, acetylsalicylic acid, also called aspirin, was synthesized by Felix Hoffman in 1897. Since then, non-steroid anti-inflammatory drugs (NSAIDs) have gradually become a mainstay in pain management. The discovery of their mechanisms was awarded the Nobel Prize in 1982. With the growing understanding of psychological factors of pain, psychologist Aaron Beck summarized the achievements and proposed cognitive behavioral therapy (CBT) in 1960s. The efficacy of CBT in treating mental disorders, including pain, has been substantiated by numerous cases. This finding underscores the tight link between pain and psychological factors. Advances in computers and algorithms have enabled rapid processing of complex data. In 1965, Melzack and Wall proposed the Gate Control theory. This theory depicted the important functions of spinal dorsal horn in modulating pain signals, offering novel insights into pain pathology and approaches to clinical pain management.^16^ A clinical trial explored the analgesic effects of spinal cord stimulation to treat eight painful patients after 2 years. Half of the patients obtained longstanding pain relief within 2 min, which first proved the superiority of spinal cord stimulation.^17^ In 1976, the opioid receptor was identified in the primate spinal cord.^18^ In the same year, Yaksh and Rudy conducted intrathecal opioid delivery of narcotics in rats. It effectively exerted potent analgesia only at the spinal level. This exploring experiment laid the foundations for the development of spinal cord stimulation therapy.^19^ In 1988, artificial intelligence (AI) was first applied in a clinical trial focusing on the pain diagnosis. The results demonstrated that AI outperformed clinicians in differential diagnosis, highlighting its potency in the pain field.^20^ Six years later, Edelman et al. utilized magnetic resonance imaging (MRI) to detect brain region activities, laying the groundwork for exploring regions involved in pain perception.^21^ At the end of 20th century, David Julius and colleagues identified the ion channel TRPV1, which is responsive to heat and then produce pain signals. This finding paved the way for discovering other temperature sensors. David Julius was honored with the Nobel Prize in 2021 for this breakthrough.

Entering new century, advanced technologies have been employed in basic research and pain management. The first clinical trial on a monoclonal antibody in neuropathic pain was reported in 2003.^22^ The effectiveness and safety of gene therapy were proved by a phase I clinical trial in 2011.^23^ A year later, the technique for converting pluripotent stem cells into nociceptors was established. This progress has provided a better in-vitro model for pain research.^24^ The associations between microbiota and pain have been revealed long before. However, it was commonly believed that microbiota activated nociceptors only through inducing inflammatory responses or secreting specific metabolites. A basic study in 2013 showed that gut microbiota could directly stimulate nociceptor neurons and induce pain sensation.^25^ The revelation shifted previous perceptions in this field and marked a milestone in microbiota and pain research. Over the last decade, research breakthroughs have continued to emerge. The organ-on-a-chip technique was applied to create a spinal microphysiological system for investigating pain and opioid-induced tolerance.^26^ It represents another significant advancement in experimental pain research tools. The latest milestone is the brain cell atlas, described using multi-omics by the BRAIN Initiative Cell Census Network project, which was reported in the special column of Science journals. This pioneering work parses brain structures at the single-cell level, providing valuable data for elucidating pain mechanisms.

## Categories of pain

Pain can be classified as nociceptive, neuropathic or nociplastic pain according to its etiology. One pain event tends to involve multiple categories. For instance, in a serious car accident, acute pain induced by open wounds can cause nociceptive pain. Spinal cord injury caused by a car crash may bring about perennial neuropathic pain. Posttraumatic stress disorder (PTSD) may also be triggered by this life-challenging event, resulting in somatic nociplastic pain. The etiology of cancer pain is more complicated, involving nerve invasion, organ damage, immune dysregulation and other unknown factors. Therefore, the clarification of pain categories is conducive to the development of pain research.

### Nociceptive pain

Nociceptive pain refers to pain induced by a physiological protective system that protects against noxious stimuli,^27^ which is the most frequent type of pain. It is by nature a transient response to actually or potentially harmful factors, triggering evasive action and protective behaviors. Inflammatory pain is one of the most representative subtypes of nociceptive pain. Somatic nociceptive pain is usually perceived in the dermis layer and is often described as lancinating, sharp or burning pain. In contrast, the sensation of visceral nociceptive pain is blurry and diffuse. The pain generated by cutting, burn and corrosion injuries can be classified as nociceptive pain.

### Neuropathic pain

Neuropathic pain is defined as pain arising as a direct consequence of a lesion or disease affecting the somatosensory system, including central neurons and peripheral fibers (Aβ, Aδ, and C fibers). According to epidemiological investigations, 7–10% of the general population experiences neuropathic pain, accounting for 20–25% of patients suffering from chronic pain.^28,29^ The prevalence of neuropathic pain is dramatically increased in individuals with specific chronic diseases due to its mechanistic particularity. Diabetic polyneuropathy, cancer, herpes zoster, multiple sclerosis and spinal cord injury are important diseases with secondary involvement in neuropathic pain. Patients with neuropathic pain typically experience a series of manifestations, such as burning and electrical-shock sensations. Persistence and poor responses to analgesics create enormous health burdens for patients, usually accompanied by psychiatric disorders, such as depression, anxiety and insomnia.

### Nociplastic pain

Some patients with explicit pain phenotypes fail to present with organic lesions and therefore cannot be classified as either of aforementioned types. In 2016, the concept of nociplastic pain was proposed and defined as a mechanistic descriptor for chronic pain states not characterized by clear activation of nociceptors or neuropathy but exhibiting clinical and psychophysical findings suggestive of altered nociceptive function. Its prevalence in the general population ranges from 5% to 15%, and there is a significant female preference.^30^ Nociplastic pain is divided into five categories: chronic widespread pain, chronic primary musculoskeletal pain, chronic primary visceral pain, chronic primary headache pain and complex regional pain syndrome.^31^ Genetic, psychosocial, and environmental factors jointly contribute to the progression of nociplastic pain.^32^

## Animal models applied for current research on pain

**Fig. 2:**
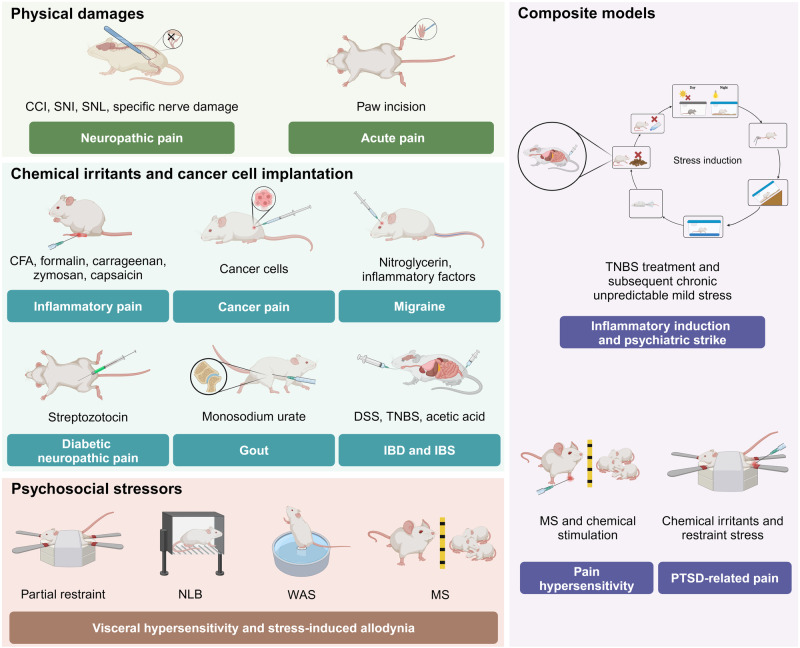
Current animal models in pain research. Physical damages, chemical irritants, cancer cell implantation and psychosocial stressors constitute the three primary methods for preparing pain models. Furthermore, composite regimens that combine several of the aforementioned methods have been employed as pain is a multifactorial event. CCI chronic constriction injury, CFA complete Freund’s adjuvant, DSS dextran sulfate sodium, IBD inflammatory bowel disease, IBS irritable bowel syndrome, MS maternal separation, NLB neonatal limited bedding, PTSD posttraumatic stress disorder, SNI spared nerve injury, SNL spinal nerve ligation, TNBS 2,4,6-trinitrobenzene sulfonic acid, WAS water avoidance stress

Experimental animal models are indispensable tools for basic and preclinical investigations into occurrence, diagnosis and treatment of pain. As pain is a multimodal event, an ideal pain model should encompass both biological and psychological factors. A diverse array of model preparation methods has been developed, including physical damages, chemical and biological irritants and psychosocial stressors (Fig. 2). Regrettably, standardized modeling approach that perfectly replicates pain development is still lacking. Most current models fail to accurately represent the mechanisms of specific pain types, potentially compromising the validity of basic research findings. In this section, we summarize the commonly employed methods of pain model generation to provide the swift access to pain research field for readers.

### Physical damages

Surgery is a common method for generating nociceptive and neuropathic pain models. Chronic constriction injury (CCI), spared nerve injury (SNI) and spinal nerve ligation (SNL) are classical approaches for inducing neuropathic pain. CCI is produced by placing loosely constrictive ligatures around the common sciatic nerve. SNI entails the incision of tibial and common peroneal nerves, sparing the sural nerve. Therefore, a key advantage of SNI is better observation of impacts of injured and non-injured nerves. Following these procedures, the metapedes of both models typically develop hyperalgesia, and the mechanical withdrawal threshold decreases. Hyperalgesia usually peaks after 7 days of surgery and persists over two months. The spontaneous ongoing pain also becomes detectable after 7 days. The spinal nerve, due to its accessible anatomical position and significant physiological functions, is another idea target. Commonly, the L5 spinal nerve, located near the dorsal root ganglion (DRG) is selected for SNL modeling.^33^ Pain perception typically develops within in 1–3 days, sooner than in CCI and SNI models. The mechanical and heat hyperalgesia can sustain 10 and 3 weeks, respectively. The spontaneous pain phenotype develops after one month of SNL.^34^ The significant advantage of SNL is better investigations into the impacts on DRG. It is noteworthy that neonatal subjects may not experience mechanical allodynia or undergo delayed-onset pain sensitivity following SNI, CCI, and SNL modeling,^35,36^ suggesting their unsuitability for early-life neuropathic pain studies. Although these three methods simulate physical nerve injury, it still remains unclear whether they can recapitulate the common diseases of neuropathic pain, such as diabetic neuropathy, neuropathic low back pain and radiculopathy.^37^ Therefore, it should be cautious to draw conclusions concerning associations between etiological factors and clinical neuropathic pain based on these models.

Given the organ and tissues specificity of innervation, some studies exploring topical pain-associated diseases involve surgical damage to specific nerves to induce hyperalgesia at targeted sites. For example, T9 laminectomy combined with radical contusion damage is used to simulate spinal cord injury.^38^ Trigeminal nerve root compression in inferior orbital fissure or inferior alveolar nerve is performed to generate animal models of trigeminal neuropathic pain.^39,40^ Furthermore, paw incision is an effective approach to imitate the status of postoperative pain or acute pain, which is extensively applied due to the simplicity and reproducibility.^41^

However, there are two significant limitations of physical damage models. First, despite precise intervention, inflammatory pain following operations, particularly in the acute phase, is inevitable. Consequently, research conclusions should be interpreted cautiously and comprehensively. Second, most methods are “all or nothing”. They lack the capability to control the extent of damage, rendering them unsuitable for studies investigating the effects of varying degrees of nerve damage, with partially different underlying mechanisms. Electrocautery tends to progress into persistent allodynia,^42^ making it more suitable for the research on pain chronicity. Additionally, electrical stimulation is also employed to trigger pain sensations. Its non-invasive nature is noteworthy. Potential inflammatory responses following invasive operations can be significantly reduced. Furthermore, some studies have verified the antalgic role of electrical stimulation.^43^ Differentiating its pain-inducing and pain-relieving effects requires further investigation.

### Chemical irritants and cancer cell implantation

Complete Freund’s adjuvant (CFA) is a water-in-oil solvent composed of mineral oil, dead *Mycobacterium tuberculosis* and an antigen salt solution. It is extensively used in preparing topically inflammatory pain or arthritis models by injection into the paw or arthrosis, respectively. Paw injection of CFA can induce pain hypersensitivity and non-evoked ongoing pain after 24 h and it will last for 1–2 weeks. Joint pain occurs after 7 days of intra-articular injection. High-dose CFA is one of few approaches to generate models at the chronic phase of pain. Furthermore, CFA elicits synovitis and bone resorption without cartilage alteration, thus it has been evaluated as a robust model for the research on rheumatic arthritis.^44^

Formalin is a protein coagulant commonly employed for tissue and cell fixation. Subcutaneous injection of formalin diluent into animal hind paws can generate local pain. Formalin-induced evoked pain and spontaneous ongoing pain are characterized by a two-phase response. The first phase (0–5 min) results from the activation of peripheral nociceptors, whereas the second phase (10–40 min) reflects the development of inflammation and central sensitization.^45^ Low-dose formalin directly activates nociceptors, while injection of high-dose formalin can exert additional tissue damage and inflammatory stimuli.^46^ Hence, the evidences indicate a significant time and dose-dependent manner of formalin-induced pain. It is usually employed for investigations into pain mechanisms. Additionally, topical injection of carrageenan is mainly used for preparing transient joint inflammation. The hyperalgesia and spontaneous nociceptive behaviors occur within 3–5 h and lasts for 24 h. Zymosan is a typical agent for acute inflammation research. It can induce thermal and mechanical hyperalgesia after 30 min in a dose-dependent manner. Spontaneous pain can be observed after 24 h of high-dose injection of zymosan.^47^ Capsaicin is commonly used for construction of skin inflammation and inflammatory bowel disease (IBD), as well as examination of analgesic drug efficacy. It promptly triggers evoked pain perception and fades within 1 h. Spontaneous ongoing pain occurs primarily within 5 min. Compared to the sustained and biphasic pain induced by formalin, it exhibits shorter lasting and monophasic duration.^48^ Intriguingly, high-dose or continuous treatment reversely lead to neuronal desensitization and analgesic effects. The modeling regimens should be carefully investigated before generating pain modeling using capsaicin.

Notably, chemical pain inducers play a crucial role in generating models of gastrointestinal disorder-associated pain. Intrarectal administration of dextran sulfate sodium (DSS) and oral treatment with 2,4,6-trinitrobenzene sulfonic acid (TNBS) are classical methods for inducing IBD. The symptoms of visceral hypersensitivity are detectable within several weeks. The pathology induced by DSS shares more features of ulcerative colitis, while the immunological and histopathological mechanisms underlying Crohn’s disease progression are following TNBS treatment.^49^ Researchers should choose proper chemical irritants according to disease types. For the research on irritable bowel syndrome (IBS), intracolonic injection of zymosan or acetic acid is commonly used, whereas with different treatment periods. Zymosan-induced visceral hypersensitivity can be detected only after 3 days.^50^ Acetic acid requires 2-week continuous treatment.^51^

Specific chemical agents are utilized for pain models based on disease-specific etiologies. For instance, diabetic neuropathy is one of the important causes of chronic pain. The streptDSSozotocin-induced diabetes model is used to investigate the mechanisms underlying neuropathic pain. The baseline of blood glucose is significantly elevated after intraperitoneal or intravenous injection of 3 days. Thermal and mechanical allodynia will be detectable after 2 weeks.^52^ The spontaneous ongoing pain lags behind evoked pain, which occurs after ~4 weeks.^53^ Vasoconstriction dysfunction and inflammatory mediators are the etiological factors of migraine. Therefore, nitroglycerin or inflammatory factors are available for the generation of migraine models. Intravenous, subcutaneous or intraperitoneal injection of nitroglycerin induces acute evoked and spontaneous pain for 3–5 h. Repeated treatment can lead to the progression of chronic pain.^54^ Topical administration of inflammatory factors, such as 5-hydroxytryptamine (5-HT), prostaglandin, histamine and bradykinin, relies on dural cannulation. Briefly, the rodents undergo craniotomy and cannula is inserted into the dura. The inflammatory factor solution is then pumped into the dura through cannula. This method can greatly avoid the effects of systemic delivery on other organs by nitroglycerin treatment, increasing the reliability of migraine models, however with a significant increase in operation complexity. Infusion numbers are positively correlated with pain hypersensitivity and duration. Three-time infusion-induced hypersensitivity sustains ~1.5 h and eight-time infusion contributes to the prolongation into more than 5 h. However, this method cannot initiate long-period spontaneous pain.^55^ Intraarticular injection of monosodium urate is a standard method for gout models. The swelling, mechanical hyperalgesia and ongoing pain can be observed after 2 h of injection. The symptoms reach the peak at 24 h and persist until 48 h.^56,57^

The mechanisms and treatment of cancer pain are hot topics of basic studies. Schwei et al. reported a bone cancer pain model.^58^ This research has sparked an upsurge in research on pain associated with various types of cancer. This technique involves local transplantation of cancer cells, with pain hypersensitivity and spontaneous pain detectable within 1–2 weeks.^59^ The concrete time varies based on cancer types, tumor cell loads and locations. Moreover, certain chemotherapeutic drugs for cancer treatment have significant neurotoxicity and easily cause neuropathic pain, such as oxaliplatin and paclitaxel. Administration of chemotherapeutic drugs can lead to phenotypes of hyperalgesia and spontaneous pain within 1 week.^60^

### Psychosocial stressors

Psychological and social factors are integral in pain perception, leading to the creation of animal models through psychosocial impairment techniques. These methods are employed to simulate clinically stress-associated diseases, such as IBS, gastric hyperalgesia and IC/BPS. For instance, partial restraint, a nontraumatic stress model, refers to wrapping the upper part of the animal’s trunk in paper tape for several hours daily. The subjects develop spontaneous pain within a couple of weeks. However, this method is unsuitable for neonatal investigations into early-life stress. Neonatal limited bedding (NLB), which refers to subjects housed in a parochial cage, serves as an early-life stressor. Subjects will endure persistent somatic hyperalgesia.^61^ Water avoidance stress (WAS) is another method to achieve movement restraint. Mice are placed on a small platform inadequate for standing by all fours and surrounded by water, which forces the subjects to remain continuously vigilant, resulting in a strong stress response. Repeated WAS can induce typical visceral hypersensitivity. Nevertheless, there have been no studies examining the effects of NLB and WAS on spontaneous ongoing pain.

The social relationship damage is also employed in pain model generation. Maternal separation (MS) is the severance between juvenile individuals and their dependent subjects, which affects nervous system development and increases the risks of adult psychiatric disorders.^62^ Therefore, MS has also been conducted in basic research on nociplastic pain. Current studies using this method have focused on visceral hypersensitivity. Animals undergoing early-life MS will suffer from pain hypersensitivity at the adult phase. Separation time is a critical factor in the effects of MS. Brief separation, more parallel to mother scavenging for food, has a relatively mild impact on juvenile subjects, whereas severe anxiety behaviors are observed following long-term separation. Likewise, the studies focusing on MS and spontaneous ongoing pain is still lacking.

### Composite models

Despite the apparent pain-inducing effects of the above single-factor models, their limitations on disease reducibility are obvious due to the etiological complexities of pain in patients. Some studies tried to simultaneously use several methods to corroborate each other.^63,64^ Furthermore, the comprehensive modeling strategies based on existing approaches can maximize the simulation capabilities of pain models. For example, the TC-IBS method includes trinitro-benzene-sulfonic acid treatment and subsequent chronic unpredictable mild stress, with properties of both inflammatory induction and psychiatric strike.^65^ Similarly, MS and chemical stimulation have been assembled to prepare a pain hypersensitivity model.^66^ In the research on PTSD-related pain, chemical irritants and restraint stress are used simultaneously. Nervous system homeostasis and neurologic functions are typically disrupted, akin to PTSD symptoms.^67^ Although these models are based on the superposition of different factors, they offer valuable insights for developing more scientific models for pain research.

## Basic circuits of pain

**Fig. 3:**
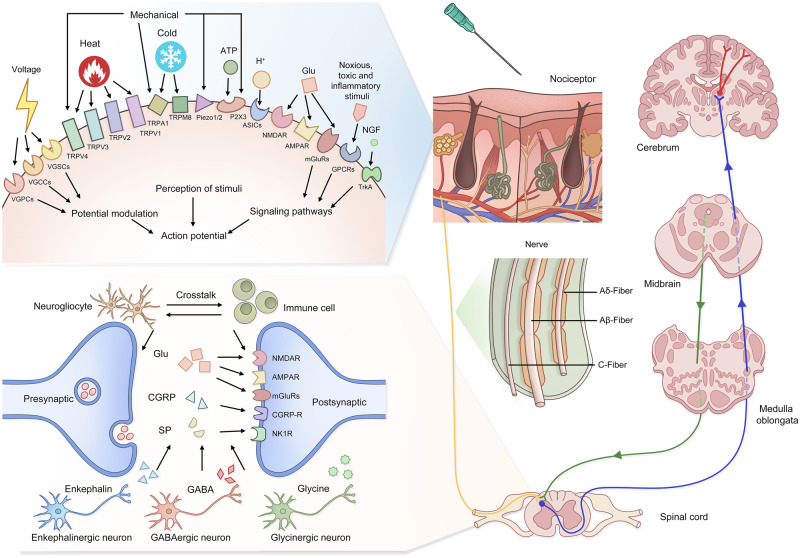
Schematic illustration of pain sensation pathways. The exposure to pain-inducing events changes activity of specific receptors and activates action potential of peripheral nociceptors. The signals are then transmitted from DRG located to the spinal cord via afferent nerves. The nerves are categorized into Aβ, Aδ and C fibers. During neuronal transmission, the presynaptic membrane releases various neurotransmitters into the subsynaptic membrane, inducing potential alterations in the subsequent neuron. The figure shows some representative neurotransmitters in during pain perception. Additionally, neurogliocytes, immune cells and other types of neurons collaboratively modulate pain signals. The DRG, as the relay station, is responsible for ascending transmission to the corresponding sensory cortex, which modulates the ultimate pain sensation. The descending regulatory pathways also play a role in pain modulation. ASIC acid-sensing ion channel, AMPAR α-amino-3-hydroxy-5- methylisoxazole-4-propionate receptor, CGRP calcitonin gene-related peptide, GABA gamma-aminobutyric acid, GPCR G protein-coupled receptor, mGluR metabotropic glutamate receptor, NGF nerve growth factor, NMDAR N-methyl-D-aspartate receptor, P2X3 purinergic receptor 3, TrkA tropomyosin-related kinase A, TRPA1 transient receptor potential ankyrin 1, TRPM8 transient receptor potential melastatin 8, TRPV1 transient receptor potential vanilloid 1, TRPV2 transient receptor potential vanilloid 2, TRPV3 transient receptor potential vanilloid 3, TRPV4 transient receptor potential vanilloid 4, VGCC voltage-gated calcium channel, VGPC voltage-gated potassium channel, VGSC voltage-gated sodium channel

Pain perception is a complex physiological process involving both the central nervous system (CNS) and peripheral nervous system (PNS). Numerous nervous structures, cells and molecules collectively underlie the transduction, transmission, modulation and perception of pain signals (Fig. 3). This section provides an overview of the basic mechanisms of pain perception for readers to better understand the subsequent contents in this review.

### Peripheral transmission of pain signals

Nociceptors, peripheral transducers of pain signals, are located in the skin, mucosa, muscles, surface and interior of tendons, periosteum, vasculature, and internal organs. They are morphologically free or undifferentiated nerve endings, the cell bodies of which reside in the DRG and trigeminal ganglion. According to the received noxious stimuli, they can be divided into thermo-sensitive, mechanical-sensitive and injury signal-sensitive types. Compared to other sensors, the activation thresholds of nociceptors are relatively higher, ensuring that human body perceives normal tactile information without pain. Nociceptors are regarded as the gatekeepers and initiators of pain sensation.

The peripheral terminals of nociceptors have many types of ion channels, which can perceive external stimuli, code signals and generate membrane excitability. Ion channels produce electrical signals through regulating the ion current across membranes. The adjacent voltage-sensitive channels are forced open in a chain reaction. According to the precipitating factors of channel opening, they can be generally divided into two categories, voltage-gated ion channels and ligand-gated ion channels.

Voltage-gated ion channels refer to a kind of transmembrane proteins whose conformation is determined by membrane potentials. They play a crucial role in converting receptor potentials into a series of action potentials. Voltage-gated sodium channel (VGSC) family comprises 9 members, including Nav1.1 to Nav1.9. VGSCs rapidly adopt open conformations following cell membrane depolarization, allowing sodium to flow into cells down a concentration gradient. This process initiates action potentials and produces pain signals at nerve endings. VGSCs have typical differences in species, spatial and temporal distributions, as well as electrophysiological characteristics.^68,69^ Nav1.7, in particular, has garnered significant attention. Mutations in the Nav1.7 encoding gene *Scn9a* are associated with various pain disorders, such as inherited erythromelalgia, paroxysmal extreme pain disorder and small-fiber neuropathy.^70^ Inhibiting Nav1.7 functions effectively mitigates neuropathic pain and stimulates the production of endogenous opioids.^71^ The role of Nav1.7 varies with different types of pain. For instance, it contributes to the development of neuropathic pain, whereas bone cancer pain and oxaliplatin-induced pain do not depend on Nav1.7-postive nociceptors.^72^ Other VGSCs, like Nav1.1, Nav1.6, and Nav1.8, also play important roles in pain modulation.^68,73,74^

Voltage-gated calcium channels (VGCCs) are distributed in all types of excitable cells. They are composed of four subunits: α1, β1-4, α2δ1-4, and γ1-8. Each VGCC type has a unique subunit composition, with α2δ being a crucial component. α2δ interacts with α1 and β subunits, enhancing peak potentials and rates of channel activation and inactivation. Noxious stimuli can upregulate α2δ expression in both the CNS and PNS, subsequently augmenting pain signals.^75,76^ The functions of calcium channels in sensory neurons are finely tuned by various factors, like adiponectin, neuromedin B and non-coding RNAs.^77–79^

In contrast to VGSCs and VGCCs, voltage-gated potassium channels (VGPCs) primarily facilitate potassium outflow from neurons, inducing membrane hyperpolarization and neuronal excitability attenuation. Noxious stimuli, such as mechanical force, heat and algogens, can downregulate potassium channel expression and inhibit their activity,^80^ leading to ectopic spontaneous discharges in nociceptors.^81^

Transient receptor potential (TRP) channels, the most representative ligand-gated ion channels, are extensively distributed in both the CNS and PNS. TRP family members act as molecular sensors of pain and itch, responding to physical and chemical stimuli. Currently, 28 TRP members have been identified, with well-documented biological functions for TRPV1, TRPV2, TRPV3, TRPV4, TRPA1, and TRPM8.^82^ The mechanistic associations of TRPV1 and TRPA1 with pain modulation have been largely investigated. Their activation states and expression levels are positively associated with pain sensation.^83,84^ Intriguingly, variouNMDARs natural biotoxins induce pain perception just through targeting TRPV1 and TRPA1,^85,86^ demonstrating the ingeniousness of interspecies evolution. The TRP channel antagonists, like V116517 and BCTC, have shown significant potential in pain management.^87,88^

N-methyl-D-aspartate receptors (NMDARs), consisting of various GluN subunits, are particularly sensitive to mechanical stimulation. Calcium influx through NMDARs is a critical inducer of electrical signal activation.^89^ NMDARs interact with calcium channel subunit α2δ, tonically activating primary afferent neurons.^90^ The ion-specific permeability is controlled by Mg^2+^, and neuronal depolarization contributes to the activation of NMDARs. Both presynaptic and postsynaptic NMDARs modulate excitatory synaptic transmission and CNS synaptic plasticity, facilitating hyperalgesia.^91,92^ Neuropathic pain persistently activates NMDARs under continuous endogenous glutamate stimulation.^93,94^ This vicious cycle further exacerbates pain chronicity .

A variety of other channels distributed in nociceptors mediate the transformation of noxious stimuli into electrical signals. The calcium-permeable purinergic receptor (P2X) channels, another class of receptors for pain sensitization, exhibit hyperactivity dependent on extracellular ATP released from damaged cells.^95^ Acid-sensing ion channels (ASICs), members of the epithelial sodium channel/degenerin family, detect alterations in extracellular pH and mediate hyperalgesia during the progression of inflammation, ischemia and cancer metastasis.^96^ ASIC3 unilaterally suppresses P2X3 receptor currents through forming the protein complex due to their similar molecular structures and cellular colocalization,^97^ illustrating the molecular mechanisms underlying pain harmonization. The identification of other receptors, such as Piezo 1/2, bradykinin and nicotinic acetylcholine receptors, has further expanded the understanding in pain generation mechanisms.^98^

In addition to ion channels, signaling transduction receptors likewise receive stimuli and modulate pain-related signaling pathways. G protein-coupled receptors (GPCRs), the largest family of transmembrane proteins, mediate the physiological control of nociceptive transmission. GPCRs are widely expressed in neurons and other pain-associated cells, like glial and immune cells. Various pain-related ligands, such as opioids, glutamate, bradykinin, gamma-aminobutyric acid (GABA), cannabinoids, 5-HT, prostaglandins and histamine, have been identified. Activation of GPCRs leads to conformational changes in the α subunit, which regulates the activity of adenylate cyclase and phospholipase C, influencing the production of secondary messengers.^99^ The mechanisms exert global effects on pain events, involving neuronal excitability, inflammatory response, intercellular communication and neurotransmitter release.^100^ The functions of some ion channels are under the rigid control of GPCR-mediated signaling.^101^ Additionally, tropomyosin receptor kinases are receptors for neurotrophic factors such as nerve growth factor (NGF) and brain-derived neurotrophic factor (BDNF), responsible for neuronal survival and growth, immune homeostasis and neurotransmitter selection.^102^

The electrical signals produced by nociceptors are subsequently transmitted to spinal cord via nerve afferent fibers, which are essentially axons of sensory neurons. There are three main categories of primary afferent fibers according to anatomical characteristics and functions, including Aβ, Aδ and C fibers. Spatial transcriptomics data further link the sensor subtypes with these nerve fibers.^103^ Nociceptors pertain to Aδ and C fibers; effective stimulation to Aδ fibers induces sharp, needle-like fast pain, while C fiber activation leads to burning, blunt or inaccurately localized pain. A-type nerve fibers are wrapped with the myelin sheath composed of Schwann cells, accelerating transmission of action potentials. Recent studies have uncovered that Schwann cells respond to noxious stimuli and elicit intracellular oxidative stress reactions. The paracrine release of reactive oxygen species, like 4-hydroxynonenal and H_2_O_2_, significantly stimulates TRPA1, sustaining pain perception.^84,104^ Additionally, Nav1.7 is required for the initiation of action potentials in C fibers.^71^ Injury and inflammation can cause nerve fiber sensitization, decreasing thresholds and inducing persistent pain experiences.^105,106^

### Ascending pain transduction tracts

A single noxious event can activate multiple neurons in the DRG. Spinal projection neurons receive, integrate and transmit the complex signals into ascending pathways, thereby completing the conversion from the PNS to the CNS.

The spinothalamic tract and the trigeminothalamic tract are two fundamental pathways of somatalgia transduction. Specifically, the spinothalamic tract is shunted into lateral and anterior branches at the anterior white commissure. The lateral spinothalamic tract is responsible for transmitting pain information from the torso and limbs, while the trigeminothalamic tract consists of fibers from the spinal trigeminal nucleus and most pontine trigeminal nuclei, conveying pain sensations from the head and face. Additionally, there are other ascending pathways for somatalgia transduction, such as the spinomesencephalic tract, spinoreticular tract, spinohypothalamic tract and dorsal column postsynaptic fiber bundles.

In contrast, pathways for visceral pain transduction are more decentralized and do not conform to classical neural circuits. The cell bodies of afferent visceral sensory neurons through sympathetic nerves are located in the T1-L3 spinal ganglion. Those via parasympathetic nerves are distributed in the sensory ganglia of the glossopharyngeal nerve and vagus, as well as in the S2-4 spinal ganglion. The axons of afferent visceral sensory neurons are distributed throughout internal organs and their blood vessels.

Action potentials induce the release of various excitatory neurotransmitters and neuromodulators derived from neurons with different functions. They collectively bind to postsynaptic receptors, activating the excitability of next-level neurons. Glutamate, a typical excitatory neurotransmitter, is produced by excitatory neurons. Its release directly induces an excitatory postsynaptic potential. Conversely, inhibitory neurons secrete specific neurotransmitters, such as GABA, glycine and opioids, to prevent neuronal overexcitation. The identified neurotransmitters are listed in Table 1. Neuromodulators do not directly trigger alterations in postsynaptic biological effects. They mainly regulate neurotransmitter release and neuron excitability mainly through binding to GPCRs and rearranging intracellular signaling pathways. For instance, BDNF, has dual functions in pain signal modulation in both the PNS and CNS.^107,108^ Calcitonin gene-related peptide (CGRP) has properties of the enhancement in presynaptic glutamate transmission and responses to substance P.^109,110^

**Table 1:** The summary of neurotransmitters involved in pain sensation

Neurotransmitter	Type	Receptor	Distribution	Function
Glutamate	Amino acid	NMDAR, AMPAR, mGluR	CNS, PNS	Excitatory
Aspartate	Amino acid	NMDAR, AMPAR, Kainate receptor	CNS	Excitatory
Histamine	Monoamine	H1	CNS, PNS	Excitatory
CGRP	Peptide	Heterotrimers of CALCRL, RAMP1 and RCP	CNS, PNS	Excitatory
Substance P	Peptide	NK1, NK2, NK3	CNS, PNS	Excitatory
GABA	Amino acid	GABA_A_, GABA_B_	CNS, PNS	Inhibitory
Glycine	Amino acid	GlyR	CNS	Inhibitory
Endogenous opioids	Peptide	μ-, κ- and δ-receptors	CNS, PNS	Inhibitory
Cannabinoids	Lipid	CB1, CB2	CNS, PNS	Inhibitory
5-HT	Indole derivative	5-HT1-4, 7	CNS, PNS	Dependent on 5-HT receptors
Norepinephrine	Monoamine	α1, α2, β adrenergic receptors	CNS, PNS	Excitatory (α1, β), inhibitory (α2)
NO	Gasotransmitter	Diffusion across membrane	CNS, PNS	Excitatory or inhibitory

5-HT 5-hydroxytryptamine, AMPAR α-amino-3-hydroxy-5- methylisoxazole-4-propionate receptor, CALCRL calcitonin-receptor-like receptor, CB cannabinoid receptor, CGRP calcitonin gene-related peptide, CNS central nerve system, GABA gamma-aminobutyric acid, GlyR glycine receptor, mGluR metabotropic glutamate receptor, NK neurokinin, NMDAR N-methyl-D-aspartate receptor, NO nitric oxide, PNS peripheral nervous system, RAMP1 receptor activity modifying protein 1, RCP CGRP-receptor component protein

Inflammatory cells (mastocyte, neutrophil, microglia, etc.) and mediators (TNF-α, interleukins, prostaglandin E2, etc.) act on neurons and affiliated cells within the nervous system. The activation of inflammatory cells and ectopic release of proinflammatory factors sensitize nociceptors and reduce pain threshold, facilitating responses to noxious stimuli and pain sensation caused by innocuous stimuli, like tickling.^111–113^ Long-term inflammation alters nervous system plasticity and promotes pain chronicity.^114^ The effects of neutrophils are time-phase dependent. Acute activation of neutrophil accelerates pain resolution. Early treatment with NSAIDs to inhibit inflammatory responses conversely prolongs the course of pain perception.^115^ Long-term infiltration of neutrophils leads to the exaggeration of nociceptive responses.^116^

Recent studies have demonstrated the impact of interactions between inflammatory cells and the nervous system on pain perception. For example, astrocytes can directly enhance microglia activity and promote pain perception.^117,118^ Lipocalin-2, an important secretory factor derived from activated astrocytes, also elevates TRPV4 expression and further promotes microglia activation.^113^ Macrophages secrete proinflammatory factors like IL-33 and recruit neutrophils. The inflammation-inducing effects of neutrophils can be promoted by IL-33, facilitating DRG excitability via TRPV1 channel activation.^56^

### Pain perception and modulation in the brain

The cerebrum coordinates the crosstalk among afferent axons, interneurons and projection neurons. A great number of clinical neuroimaging studies and basic research have identified a “pain matrix” in the brain, including a variety of brain nuclei like the periaqueductal gray (PAG), thalamus, hypothalamus, parabrachial nuclei (PB), nucleus tractus solitarius, amygdala, insular cortex (IC), somatosensory cortex, anterior cingulate cortex (ACC), prefrontal cortex (PFC). These areas appear to collaboratively modulate pain signals. The primary somatosensory cortex (S1) is a central hub of noxious sensation, receiving thalamocortical input from the ventral posterolateral thalamus.^119^ The output signals from S1 are transmitted to thalamic nuclei and several subcortical targets.^120^ The neuronal activity and connectivity in S1 are dramatically enhanced following pain signal transmission, with neurons in layer 6 amplifying thalamocortical signaling while inhibiting innate antinociceptive mechanisms.^121^ Furthermore, the lateral PB and thalamus are key areas in receiving nociceptive projections from the spinal cord and integrating competitive signals that modulate pain. Chronic pain promotes significant changes in sensory circuit reorganization and metabolic patterns of these brain regions,^122,123^ further facilitating central sensitization. Along with PB, the central amygdala (CeA) also mediates pathophysiologic effects and behavioral responses to noxious stimuli through neural circuits connecting with brainstem and hypothalamus.^124,125^

Pain perception depends not only on the damage degree but also on emotional, social and environmental factors. The degree of attention paid to pain, cognitive appraisal of threats and individual character are all associated with pain perception. Depressive states may aggravate or even directly elicit pain.^126^ Pain memory can also reproduce forepassed pain feelings and cause hyperalgesia.^127^ In terms of brain regions involved in emotion modulation, areas like S1, IC, ACC and PFC process afferent signals to generate pain perception and affection.^128^ Particularly, the medial PFC (mPFC) plays a critical role in the development of chronic pain.^129^ A subgroup of specific neuronal ensembles in the dorsomedial PFC processes nociceptive information and regulates pain chronicity.^129^ Two implicated clusters of PFC neurons project to limbic regions, including the hypothalamus, nucleus accumbens (NAc) and amygdala, underpinning the negative emotional and physiological impacts on chronic pain.^130^ Dopaminergic pathways in the ventral tegmental area and the substantia nigra compacta-NAc projection are responsible for pain aversion and pain-relief reward modulation.^131^ In summary, many important neural circuits contribute to the development of pain comorbidity.

Apart from the traditional pain circuits, many other areas are implicated in the perception and modulation of pain. For example, the entorhinal cortex and medial septum to hippocampus circuit, typically recognized for their roles in learning, memory or emotion regulation, have been found to modulate pathological pain.^132–134^ A contribution from adult hippocampal neurogenesis underlies pain chronicity, as well as the alleviative effects of environmental enrichment and exercise on chronic pain.^135,136^

In addition to the ascending pain transduction tracts, many brain areas in the descending pain modulation pathways are involved, such as the PAG, rostral ventromedial medulla (RVM), locus coeruleus, lateral reticular nucleus, nucleus raphes magnus, nucleus reticularis paragiagantocellularis. These pathways are classified as descending inhibitory and facilitatory systems. They collectively keep a subtle balance under normal conditions. The development of pain hypersensitivity can disrupt this balance, favoring the descending facilitatory system.

The PAG, RVM and a portion of the pontine dorsolateral reticular formation participate in the descending inhibitory system. These brain areas exert inhibitory modulation on nociceptive information via the descending spinal dorsolateral tract. Multiple nuclei have their own descending pathways towards spinal dorsal horn. The PAG is a hub of descending inhibitory system, with the dorsomedial PFC, amygdala and hypothalamus regulating its activation.^137–139^ The projection from the PAG to RVM and locus coeruleus attenuates neuropathic pain and accompanying emotional dysregulation.^140,141^ The RVM receives input signals from the PAG, nucleus tractus solitaries, parabrachial nucleus and other supraspinal sites processing nociceptive information. Two types of functional neurons, On-cell and Off-cell, have been identified in the RVM, executing descending facilitation and inhibition, and collaboratively determining the adjusting strengths of the RVM.^142^

The descending facilitatory system enhances responses to noxious stimuli by decreasing pain thresholds. It is not typically active during normal pain perception. Pain hypersensitivity and catastrophizing can suppress the activity of the inhibitory system while arousing the facilitatory system.^143,144^ The RVM, ACC, PAG, parabrachial nucleus are major components of this system.^145^ Despite distinct regulatory functions, it is not difficult to find that there is considerable overlap in anatomical regions. The bidirectional functions of the RVM have been greatly manifested, possibly attributable to different subtypes of neurons in the nuclei. Descending facilitation from this region is the critical factor of neuropathic pain development.^146^ Furthermore, physiological behaviors are partially governed by the limbic system, which modulates activities of the brain regions in descending facilitatory system.^147^ This is an essential mechanism by which pain perception is affected by individual emotion, experience and memory.

Default mode network (DMN) is a collection of brain regions that are actively engaged when an individual is at rest or not actively engaged in a task that requires external attention. DMN mainly includes the precuneus, posterior cingulate cortex, mPFC, medial temporal lobe and angular gyrus. These regions are interconnected and work together to support internally focused cognition, including self-reflection, episodic memory retrieval, and mind-wandering. DMN is implicated in the regulation of pain sensation. Pain competes for cognitive resources with other kinds of attention-demanding stimuli, which is closely associated with DMN dysfunctions. The functional connectivity between brain regions within DMN is enhanced and its amplification is correlated with pain severity.^148^ Specifically, mPFC, a critical hub of DMN, can present increased high frequency oscillations. Its connectivity to the posterior constituents of the DMN is meanwhile impaired.^149^ In the patients with persistent post-traumatic headache, PAG-seeded functional connectivity is reduced, accompanied by the structural reconstruction of the ACC and posterior cingulate cortex.^150,151^ In addition to internal networks, the abnormality of DNM connectivity with other brain regions has been extensively discovered, involving the insula, ventral lateral/posterolateral nucleus and postcentral gyrus.^152–154^ The advances in DMN research provide strong proof for identifying mechanisms underlying emotional changes that affect pain perception. For instance, mind wandering restores the ectopic connectivity between PAG and DMN, redirecting spontaneous attention away from pain.^155^ The thalamic-DMN decoupling has been proved as an important mechanism of mindfulness meditation.^156^ On the other hand, negative mood promotes pain hypersensitivity through influencing DMN functional connectivity during the progression of chronic pain.^157^ Notably, despite close associations between DMN and chronic pain shown by most studies, acute pain can likewise induce alterations in oscillatory activity and functional connectivity of DMN, which underpins attentional processes in the presence of pain.^158^

## Molecular mechanisms of pain modulation

**Fig. 4:**
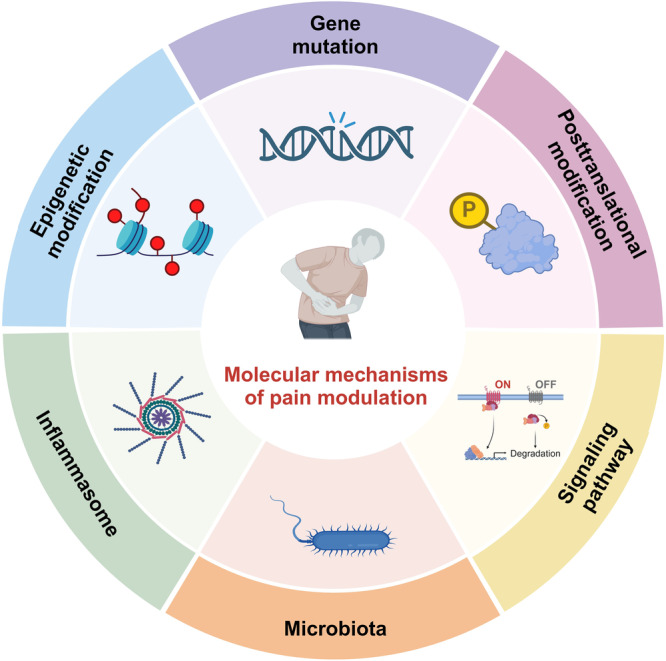
The schematic illustration of molecular mechanisms underlying pain modulation. The molecular mechanisms are generally categorized into six aspects, including gene mutation, epigenetic modification, posttranslational modification, inflammasome, signaling pathways and microbiota. They orchestrate pain perception and modulation

In addition to the basic circuits and corresponding molecules as introduced above, a series of molecular mechanisms underlie pain perception under intricate but well-regulated control. With the development of high-quality preclinical research, the scattered advancements are gradually converging into the systemic body of knowledge, contributing to the identification of numerous promising therapeutic targets. Herein, we summarize current achievements in related molecular mechanisms to present a more complete network of pain modulation (Fig. 4).

### Gene mutation

Most gene mutations are neutral, but a small minority may cause diseases, including pain disorders. Various mutations can lead to totally different clinical outcomes, ranging from pain insensitivity to extreme pain sensation. Erythromelalgia, familial episodic pain syndrome, congenital insensitivity to pain with anhidrosis and Fabry disease are the representative inherited diseases with specific gene mutations. Due to the individual differences, mutation patterns associated with pain disorders are sporadic and most data have been presented as case reports. Mutations in ion channel-encoding genes account for a large portion of existing investigations.

Mutations of multiple sites of *Scn9a* gene cause truncation or function loss of Nav1.7, leading to congenital insensitivity to pain. Some cases are complicated with anosmia, while other patients have normal olfactory sensation,^159–161^ suggesting that mechanisms by which Nav1.7 modulates pain and olfaction partially overlap. Common missense mutants of *Scn9a* are correlated with pain severity of clinical patients with symptomatic disc herniation.^162^ Mutations in introns, which do not directly encode Nav1.7 protein, can also affect pain sensitivity. A novel homozygous substitution in *Scn9a* intron 3 interferes with mRNA splicing and leads to Nav1.7 inactivation. Furthermore, mutations are not confined to channel function deficiency. A1632E is a type of gain‐of‐function mutation. Nav1.7/A1632E mutants can form dimers and maintain persistent currents, exempt from the effects of inactivation particles targeting VGSCs.^163^ Such non-canonical mechanisms greatly expand the understanding in gene mutation functions.

Mutations in other VGSC-encoding genes also contribute to the dysregulation of pain perception. Two missense mutations in *Scn11a* (c.673 C > T and c.2423 C > G) facilitate channel activity and promote hyperexcitability of Nav1.9 in DRG sensory neurons, which is a critical reason for familial episodic pain syndrome.^164^ The mutation at the R222S site of *Scn11a* has also been identified in patients with mechanical hyperalgesia sensitive to cold exposure.^165^ Conversely, a heterozygous nonsynonymous mutation in exon 15 of *Scn11a* causes excessive activation at resting potential and sustained depolarization of nociceptors in individuals with the congenital inability to experience pain. The resultant action potential and excitatory transmission are impaired, leading to a loss of pain perception. This mechanism of overactivation-induced inactivation is similar to pain relief by capsaicin.^166^ Additionally, the Nav1.1 channel with L263V missense mutation enhances spike activity induced by P2X3 and 5-HT3 receptors, increasing the excitability of peripheral trigeminal neurons and contributing to migraine pain .^167^

One mutation pattern of the Cav3.1 channel in trigeminal neuralgia has been recently identified. The missense mutation of *Cacna1g* gene, encoding α1 subunit of Cav3.1, leads to the replacement of arginine with glutamine at position 706. Current density is enhanced and neuron excitability is significantly elevated.^168^ Intriguingly, an α2δ1 mutant with arginine at position 217 does not change pain sensitivity but blocks the analgesic efficacy of pregabalin for neuropathic pain.^169^ This finding clearly demonstrates that the analgesic action of pregabalin relies on α2δ1 subunit blockade.

In addition to VGSC and VGCC mutations, different VGPC variants have distinct impacts on pain sensitivity. A frameshift mutation in *Kcnk18* gene, encoding the two-pore potassium channel, causes its loss of functions. Neuronal excitability is significantly increased, exaggerating mechanical and thermal hypersensitivity during migraine progression.^170^ A recent study focusing on gene mutations in women requiring no analgesia during childbirth has identified *Kcng4* with excessive heterozygotes carrying the rare allele of SNP rs140124801. The product, Kv6.4 mutant, loses the capability of regulating Kv2.1 activity. The potassium outflux and sensory neuron hyperpolarization in uterus are promoted, attenuating childbirth pain.^171^

The roles of mutations in representative members of the TRP family in pain modulation have been unveiled. TRPV1 with N331K mutation directly causes functional deficiency.^172^ The G564S mutant is a gain-of-function variant. Nevertheless, the overactivation-induced inactivation is also observed in this mutation pattern. This membrane transport of G564S mutant is simultaneously inhibited.^173^ Notably, in addition to natural mutation, *Trpv1* gene can be chemically edited by an alkylating agent to produce a loss-of-function product.^174^ For the research on TRPA1, the N855S mutant exhibits a fivefold increase in inward current in activated nociceptors, resulting in the development of familial episodic pain syndrome.^175,176^ A nonsense mutation in *Trpa1* gene causes TRPV1 protein truncation, which can further assemble with wildtype TRPA1. The complex lowers energetic barriers and alters pore architecture, leading to neuronal hyperactivation.^177^

Additionally, mutations in genes regulating neuron development and axon outgrowth have been found to modulate pain sensation loss or sensitivity, including transcription factors, structural proteins, membrane channels and receptors.^178–182^ Taken together, a great number of genes and mutated sites have been identified to have associations and causalities with pain. However, we have to acknowledge current research limitations: i) The concrete mechanisms by which these mutated proteins gain or lose functions are largely unknown. High-resolution structures and interactions may be promising research directions. ii) The typical individual differences in gene mutations mean that current achievements have lower universality, limiting their further clinical translation. iii) Few studies have investigated potential drugs targeting the mutants, leading to the dreadful scarcity of clinical therapies against congenital pain disorders. Robert et al. found a peptide with properties of blocking P2X7 receptor mutants without restraining normal channels, which is associated with nerve injury and inflammatory allodynia.^183^

### Epigenetic modification

**Fig. 5:**
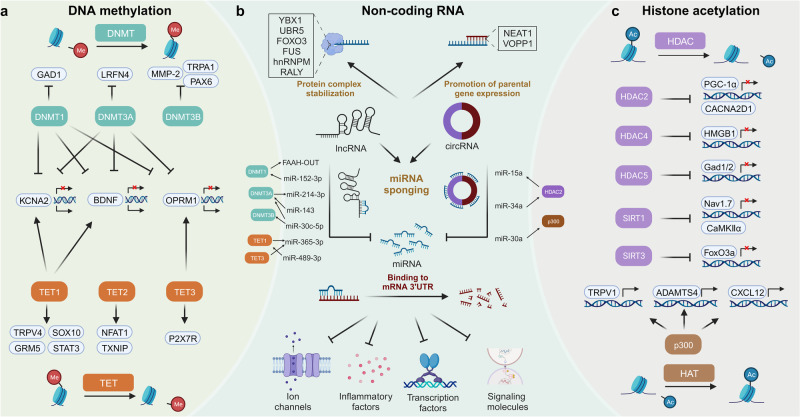
The mechanisms of epigenetic modification in pain modulation. The mechanisms are categorized into three aspects: DNA methylation, non-coding RNA and histone acetylation. **a** For DNA methylation, DNMTs and TETs are responsible for DNA methylation and demethylation, respectively. They regulate expression of various genes associated with pain perception. The expression of KCNA2, BDNF and OPRM1 are simultaneously under the control of DNMTs and TETs. **b** Non-coding RNAs, comprising miRNAs, lncRNAs and circRNAs, play various roles. miRNAs can bind to 3’UTR of mRNAs associated with pain, negatively regulating their expression. Some lncRNAs and circRNAs act as miRNA sponges to counteract the functions of downstream targets. Certain lncRNAs and circRNAs directly interact with proteins to enhance their stabilization, thereby affecting pain sensitivity. Several non-coding RNAs, like lncRNA NEAT1 and circVOPP1, have been shown to stabilize the mRNAs of their parental genes related to pain to promote their expression. **c** HDACs and HATs collaboratively maintain the balance in histone acetylation. Specific HDACs, including HDAC2, HDAC4, HDAC5, SIRT1 and SIRT3, along with HAT p300, regulate expression of genes involved in pain modulation. Notably, non-coding RNAs regulate expression of enzymes associated with DNA methylation and histone acetylation. The expression of non-coding RNAs are, in turn, regulated by the other two mechanisms. circRNA circular RNA, DNMT DNA methyltransferase, HAT histone acetyltransferase, HDAC histone deacetylase, lncRNA long non-coding RNA, miRNA microRNA, TET ten-eleven-translocation protein, UTR untranslated region

Despite differences in hereditary information, there are extremely high similarities in gene sequences between individuals with significantly different characteristics. Environment, behavior and age can produce apparent and persistent influences on humans. These phenomena cannot be forcefully explained by inherent genetic information alone. Epigenetics refers to alterations in gene expression not rooted in DNA sequences. Rapid advancements in epigenetics knowledge have unveiled novel mechanisms underlying physiological and pathological processes. It primarily includes DNA methylation, histone modification and non-coding RNAs. These three molecular mechanisms play essential roles in pain modulation (Fig. 5).

DNA methylation pertains to forms of DNA chemical modification. Catalysis of DNA methyltransferases (DNMTs) can transfer methyl groups derived from S-adenosylmethionine to specific bases. Most DNA methylation sites exhibit aggregated distributions, known as CpG islands. DNA methylation changes chromatin structure, DNA conformation, DNA stability and interactions with proteins, precisely regulating gene expression without editing base sequences. Studies have shown close associations between DNA methylation and pain perception.

Patients suffering from chronic pain universally undergo significant changes in DNA methylation states, particularly in promoter regions.^184,185^ Global methylation data have been used to investigate pain-associated mechanisms with the support of bioinformatic analysis, such as G-protein coupled cholinergic signaling, neuron development and immunomodulation.^184,186,187^ DNA methylation has quick responses to pain. Its alterations can be detected at the early phase of neuropathic pain and persist chronically.^188^ DNA methylation has disease and organ specificities. For example, there are huge differences between DNA methylation induced by diabetes neuropathy, nerve injury and chemotherapy, although they are all typical neuropathic pain. The CpG sites present prevailing hypomethylation in DRG, whereas the CNS, such as spinal cord and PFC, gains more DNA methylation.^187–189^ The methylation levels of genes encoding classical positive regulators of neuropathic and nociplastic pain sensation, like TRPA1, CGRP, and BDNF, are significantly altered in patients with pain disorders. The methylation levels negatively regulate their expression, potentially causing hyperalgesia or pain insensitivity.^190–193^

The DNMT family mainly consists of three enzymes with catalytic activity, including DNMT1, DNMT3A, and DNMT3B, responsible for adding methyl to specific gene regions. They generally present hypomethylation and participate in neuropathic pain modulation in both the CNS and PNS.^194^ DNMT1 and DNMT3A upregulate methylation of promoter and 5’-untranslated region of *Kcna2* gene, decreasing membrane densities of VGPCs and Kv current, leading to central sensitization and neuropathic pain.^194,195^ They also methylate promoters of genes encoding non-coding RNAs, with dysfunctions in these downstream non-coding RNAs contributing to various pain disorders, ranging from pain hypersensitivity to insensitivity.^196,197^ Systemic inhibition of DNMT activity results in alleviation of neuropathic pain.^185^ Therefore, it can be concluded that despite manifold targets of DNA methylation, its overall effects are pain hypersensitivity.

Ten-eleven-translocation proteins (TETs) mediate DNA demethylation, dramatically maintaining DNA methylation stability and shaping epigenome landscape along with the DNMT family. TET1, TET2, and TET3 are the main members. The double-sided nature of TET1 has been revealed. On the one hand, it can remove restrictions on gene expression induced by DNA methylation during the progression of nociceptive and neuropathic pain, involving membrane receptors (mGluR5), ion channels (TRPV4), transcription factors (SOX10), and signal transduction factors (STAT3 and BDNF).^198–201^ On the other hand, some reports have shown the analgesic properties of TET1. It can rescue suppression of VGPC functions by regulating methylation of *Kcna2* and *K2p1.1* promoters in the neuropathic pain models.^202,203^ Restoration of PROX1 levels following TET1 overexpression attenuates depression comorbidity through neurogenesis enhancement.^204^ Some studies have claimed the opposite roles of TET1 in the same therapy.^198,204^ More strangely, the contradictory data are based on investigations into the similar pain types and model generation methods, reflecting the complexity of epigenetic modification in pain sensation. Some factors not easily perceived, such as pain inducer doses, disease courses and experimental environments, may affect DNA methylation and require more attention in subsequent research.

The *Oprm1* gene encodes μ-opioid receptor and its hypermethylation positively correlates with pain severity and opioid tolerance. Long-term exposure to opioids further enhances *Oprm1* methylation levels.^205,206^ These vicious cycles via epigenomics are critical mechanisms underlying the opioid tolerance development. Moreover, molecules with properties of neuropathic and nociplastic pain modulation, such as stress-related protein FKBP5, peptide hormone leptin, CDK5 regulatory subunit-associated protein CDK5RAP1 are under strict control of DNA methylation.^207–209^

Histone is a key component of chromatin, with five types of core histones, including H1, H2A, H2B, H3, and H4. Histone acetylation, primarily occurring at lysine sites of H3 and H4, is an essential mechanism controlling histone activity. Unlike DNA methylation, acute pain has no evident impact on histone acetylation, which only responds to pain chronicity.^42^ Global alterations in histone acetylation are identified in both CNS and PNS.^39,210,211^ During nociceptive and neuropathic pain development, H3 and H4 acetylation is upregulated in DRG and spinal dorsal horn.^212^ Key brain regions, such as the CeA, PFC and hippocampus, exhibit significant changes in histone acetylation, which are involved in visceral hypersensitivity, neuropathic pain sensation and its comorbidities.^213–215^ In the descending pain modulation pathways, persistent enhancement of H3 acetylation occurs in the RVM, while this molecular event is short-lived, fading after long-term stress in the locus coeruleus.^211,216^ These findings suggest distinct regulatory effects of histone acetylation in different brain regions. Inflammatory mediators like IL-6 and TNF-α promote hyperacetylation of H3 and H4, enhancing neuron excitability in neuropathic models.^215,217^

The dynamic balance of histone acetylation is maintained by histone acetyltransferases (HATs) and histone deacetylases (HDACs). HDACs have 18 kinds of members, some of which are closely associated with pain perception. The reductions in HDAC1 and HDAC2 expression lead to the abnormal synaptic transmission, followed by somatic and visceral hypersensitivity.^75,213,218^ However, nuclear recruitment of HDAC2 driven by transcription factor Sp1 conversely aggravates neuronal dysregulation and microglial inflammation,^219^ suggesting that the cellular distribution of epigenetic regulators is another factor in pain modulation. Existing negative results concerning HDAC3 indicate its weak associations with pain modulation.^75,213^ Inhibition of HDAC4 translocation into the cytoplasm epigenetically decreases HMGB1 expression and functions as an analgesic approach for neuropathic pain.^220^ Accumulation of HDAC5 in the nucleus inhibits H3 acetylation of *Gad1* and *Gad2* promoters, impairing GABAergic neuron activity and contributing to aberrant activation of astrocytes through direct interaction with STAT3. These mechanisms can lead to the development of peripheral neuropathic pain.^221,222^ The analgesic properties of SIRT1 and SIRT3, class III of HDACs, have also been revealed. Restoring their expression downregulation in nervous lesions mitigates ectopic discharge of sensory neurons and excessive oxidative stress,^223,224^ alleviating emotional vulnerability of neuropathic pain.^214^

p300 is a representative molecule for pain modulation among HATs. Neuropathy following chemotherapy, stress and diabetes results in the upregulation of p300 expression or enhancement in p300 activity. It epigenetically modifies the hypothalamic–pituitary–adrenal (HPA) axis and promotes responses to norepinephrine.^225–227^ p300 is also involved in inflammatory pain through activating macrophages and elevating expression of TNF-α, IL-1β, CCL2, and CXCL10.^228^ Regretfully, other HATs’ roles in modulating pain are rarely investigated. Future research should pay attention to this shortcoming.

EZH2 is a histone methyltransferase catalyzing histone H3 methylation on K27 site.^229^ In the rodent models suffering from nerve injury and cancer pain, the expression of EZH2 can be significantly upregulated in the CNS. The microglia are subsequently activated, accompanied with the abrupt release of proinflammatory factors. These mechanisms contribute to the development of mechanical and thermal hyperalgesia. Downregulation of EZH2 expression or topical injection of EZH2 inhibitors have been found to alleviate neuropathic and cancer pain.^230–232^ Although several investigations have verified the pain-induced role of EZH2, its regulatory network of molecular mechanisms is still largely unclear. One study shows that mTOR signaling pathway-mediated autophagy may be a functional target of EZH2.^233^ The expression and activity of EZH2 are also under rigorous control of non-coding RNAs, including lncenc1, miR-124-3p, and miR-378.^234–236^ Moreover, EZH2 has been selected as a biomarker of evaluating efficacy of analgesic methods for neuropathic pain.^237^

Non-coding RNAs are multiple kinds of RNAs mostly incapable of encoding proteins, but their functions are not secondary to proteins. MicroRNAs (miRNAs), long non-coding RNAs (lncRNAs) and circular RNAs (circRNAs) are key molecules. Their remarkable mechanisms in pain modulation have been demonstrated by numerous studies.

The primary function of miRNAs is binding to the 3′ untranslated regions of mRNA, blocking mRNA translation and promoting mRNA degradation. Patients with pain experience have evidently altered miRNA profiles, consequently dysregulating the expression of downstream targets.^238^ A large number of target genes have been identified, including but not limited to ion channels, inflammatory mediators, signaling molecules and transcription factors.^239–243^ Importantly, the miRNA regulatory network on pain is intricate, although most studies focused on their one-to-one relationships with target genes. One miRNA can regulate many downstream mRNAs. miR-183 cluster controls expression of over 80% of recognized pain-regulated genes.^244^ One target gene is likewise under regulation by multiple miRNAs, such as TRPV1.^245,246^

LncRNAs and circRNAs can suppress miRNAs through complementary base pairing, described as miRNA sponges. This mechanism is the main research direction of current studies on pain modulation.^247,248^ Additionally, some lncRNAs and circRNAs interact with transcription factors, changing their activity and nuclear localization. These molecular events lead to alterations in neuropathic pain-related gene expression, such as KCNN1, G9A, and VEGFB.^249–251^ Certain lncRNAs and circRNAs have capabilities in regulating parental gene expression and modulating neuropathic pain, like lncRNA NEAT1 and circVOPP1.^252,253^

Non-coding RNAs as exosomal cargos play important roles in intercellular communications. Hyperactivated neurons release exosomes loaded with non-coding RNAs with immunomodulation properties, like miR-21-5p. The exosomes are phagocytosed by macrophages, initiating the proinflammatory phenotype.^254^ This evidence demonstrates that sensory neurons are not only victims, but also accomplices in the progression of hyperalgesia triggered by neuroinflammation. The interactions among astrocytes, microglia and macrophages via exosomal non-coding RNAs exquisitely regulate inflammatory pain degrees.^255,256^ Altogether, the ectopic levels of non-coding RNAs have great potential in pain evaluation.^257^ Correction of abnormal non-coding RNA networks using gene editing and chemical treatment has achieved favorable outcomes for nociceptive, nociplastic and neuropathic pain as shown in preclinical research.^258–260^ Future clinical trials are eagerly required to promote the translational application of non-coding RNAs.

Notably, there exists crosstalk between the above three aspects of epigenetic modification. For example, DNA methylation and histone acetylation jointly regulate the expression of neuropathic pain-related genes.^225,261^ miRNAs directly suppress the expression of key enzymes of the other epigenetic aspects.^239^ Noncoding RNA expression is under control of DNA methylation and histone acetylation.^225,243,262^ Overall, investigations into epigenetic modification have unveiled a new landscape of mechanisms underlying pain modulation. The achievements may lay the foundations for progress in pain management.

### Posttranslational modification (PTM)

The activity, structure, cellular localization and interactions of proteins are critically regulated by PTMs. PTMs refer to the chemical modifications involving the addition or removal of specific groups in amino acid residues. To date, more than 600 kinds of PTMs have been identified. Common PTMs include phosphorylation, ubiquitination, glycosylation, methylation, etc. Histone acetylation, as mentioned in the previous section of epigenetic modification, also belong to PTMs. Novel PTMs, such as crotonylation, succinylation and lactylation, are continuously being discovered with advancements in biotechnology.^263^ The uncovered mechanisms concerning PTMs in pain modulation are concentrated on several PTMs.

The associations between phosphorylation and pain have received the most attention among PTMs. Fyn, a member of the Src family protein kinases, phosphorylates downstream targets. Its regulatory functions on pain perception have been extensively revealed. In responses to nerve injury and inflammation, IL-33 and BDNF enhance phosphorylation and catalytic action of Fyn in a PKA-dependent manner.^264^ GluN2B, a subunit of NMDAR, at Tyr1472 is phosphorylated by Fyn. This molecular event inhibits GluN2B endocytosis, increasing its membrane densities and synaptic currents mediated by NMDAR.^264,265^ The molecular functions of SHP-1 are opposite to Fyn, mediating target protein dephosphorylation. The DRG produces PD-L1 in response to acute and chronic pain. It further phosphorylates SHP-1, downregulating expression and phosphorylation of TRPV1.^266,267^ The attenuation of sodium channels and potentiation of potassium channels mediated by SHP-1 are observed, inhibiting the excitability of sensory neurons^267,268^ However, this mechanism can also be exploited by melanoma cells secreting PD-L1, leading to pain relief in the early stages of cancer development.^267^ The inconspicuous symptoms may contribute to delayed treatment and poor prognosis in cancer patients.

From the perspective of pain-related molecules regulated by phosphorylation, ion channels and hyperalgesia-related signaling pathways are the main targets. Nav1.7 phosphorylation by PKC and Fyn,^269,270^ TRPA1 phosphorylation by CDK5^271^ and constitutive phosphorylation of TRPM8^272^ are the key regulators of pain perception. Most signaling pathways rely on phosphorylation regulation. The activities of these pathways sustain functions of neurons and non-neuronal cells. For example, the NF-κB signaling pathway, closely associated with inflammation, is activated via p65 subunit phosphorylation in neurons, astrocytes and microglia, facilitating neuropathic and endometriosis-associated pain progression.^273,274^ The MAPK signaling mediates signal transduction from the membrane to the nucleus. p38 MAPK phosphorylation promotes the development of neuropathic and postoperative pain sensation through affecting downstream molecules and crosstalk with other signaling pathways.^275,276^ MAPK phosphorylation in immune cells is crucial for regulating the release of proinflammatory factors.^277^ Moreover, STAT3, Wnt, AMPK, mTOR, JNK signaling pathways involved in pain modulation are also under control of phosphorylation.^278,279^

Ubiquitination is another modification associated with pain modulation. The classical mechanism of ubiquitination involves the degradation of endogenous proteins catalyzed by the concerted action of ubiquitin-activating enzyme (E1), ubiquitin-conjugating enzymes (E2) and ubiquitin-protein ligases (E3). NEDD4, an E3 ubiquitin ligase, promotes the degradation of multiple target proteins enhancing neuropathic pain hypersensitivity, including Nav1.7, TRPA1, and NMDAR subunit GluN2D.^280–282^ Nerve injury-induced NEDD4 downregulation is an important molecular event in pain development, regulated by histone acetylation.^281^ Restoration of NEDD4 dysregulation effectively mitigates allodynia phenotypes.^283^ Cbl-b, another E3 ubiquitin ligase, processes nociceptive information in the spinal cord. Noxious stimuli induce Cbl-b dephosphorylation.^284^ The ubiquitination levels of GluN2B and TrkA are subsequently decreased, leading to inflammatory pain hypersensitivity.^284,285^ However, some studies have reported the neuropathic pain-inducing functions of Cbl-b based on its direct ubiquitination effects on K^+^-Cl^−^ co-transporter 2 and IL-2.^286,287^ The paradoxical conclusions suggest that roles of ubiquitination enzymes may depend on pain types and progression stages.

VGCC activity is susceptible to ubiquitination. TRPV1, IL-1β and HMGB1 activate functions of the deubiquitinating enzyme USP5.^288–290^ USP5 directly interacts with Cav3.2 and potentiates whole-cell currents, promoting neuropathic and inflammatory pain development.^291^ Many follow-up studies have verified the functions of the USP5/Cav3.2 axis and explored therapeutic approaches targeting this ubiquitinating event.^292,293^ For Cav2.2, its elements in the proximal C terminus are liable to ubiquitination,^294^ which has been found to contribute to neuropathic allodynia.^295^ The roles of other ubiquitination enzymes, like HUWE1 and UBR5, in nociceptive and neuropathic pain modulation have been partially revealed.^296,297^ In the present context, all the ubiquitination enzymes linked to pain modulation pertain to E3 ubiquitin ligases.

Small ubiquitin-related modifier (SUMOylation) has similar modification processes with ubiquitination, but its mechanisms and functions have considerable differences. SUMOylation relies on covalent conjugation of SUMO with target proteins and regulates their localization, interaction and stability.^298^ Despite its late discovery of SUMOylation, its associations with hyperalgesia have garnered much attention. Dynamic alterations in SUMOylation can be detected in response to neuronal activity within a few minutes, reconfiguring ionic current densities.^299^ The robust impact of SUMOylation is ascribed to wide-range regulation of ion channels. CRMP2, coordinated by neuronal development and synaptic plasticity, is SUMOylated with SUMOylation E2 enzyme Ubc9, facilitating membrane localization and current density of Nav1.7.^300,301^ CRMP2 SUMOylation is a potential biomarker of persistent neuropathic pain.^302^ CRMP2 SUMOylation is also regulated by multiple PTMs. SUMO-specific protease SENP1 induces CRMP2 deSUMOylation, eliciting antinociceptive effects.^303^ CRMP2 phosphorylation by CDK5 enhances its SUMOylation, whereas Fyn-induced phosphorylation downregulates SUMOylation.^304^ Antagonists targeting CRMP2/Ubc9 axis have shown desirable performances in trigeminal neuropathic pain relief.^305^ Importantly, CRMP2 SUMOylation occurs in chronic neuropathic pain, rather than physiological pain.^301^ Therapies against this mechanism may have fewer side effects of losing sensations of normal acute pain. Besides CRMP2, other mechanisms by which SUMOylation affects ion channels have been identified, including direct promotion of Kir7.1 and HCN, suppression of USP5/Cav3.2 axis and enhancement in PKCε functions to phosphorylate TRPV1.^306–309^

Other PTMs likewise participate in pain development. Abnormal glycosylation is a crucial factor in diabetic complications due to dysglycemia. N-glycosylation modification enhances pore opening, channel permeability and membrane expression of ion channels like Cav3.2 and TRPV1.^310–312^ Nitrosylation, carbonylation, palmitoylation and succinylation have also been proved to modulate nociceptive and neuropathic pain perception, as well as opioid tolerance.^313–316^

In summary, PTMs constitute regulatory networks of pain modulation, exerting milder and reversible effects on protein functions compared to other mechanisms. Furthermore, PTMs can respond more quickly to noxious stimuli probably because PTMs function on the basis of mature proteins and do not require their de novo synthesis. These features underscore the prominence of PTMs in pain evaluation and treatment. Although our current understanding represents just the tip of the iceberg, the immense research and clinical value of PTMs has already emerged.

### Inflammasome

**Fig. 6:**
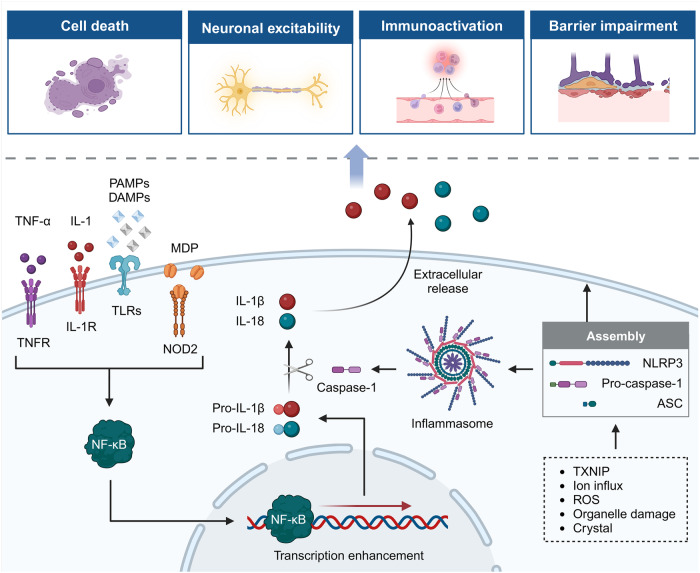
The mechanisms underlying NLRP3 inflammasome-medicated hyperalgesia. Certain ligands, including TNF-α, IL-1, PAMPs, DAMPs and MDP, bind to the corresponding receptors, activating NF-κB signaling. The activated NF-κB is transported into the nucleus and promote the expression of pro-IL-1β and pro-IL-18. Various molecular and cellular events regulate the assembly of inflammasomes. Caspase-1, derived from inflammasomes, facilitates the maturation of IL-1β and IL-18. These cytokines are subsequently released, leading to cell death, neuronal hyperexcitability, immune activation and impairment of brain barriers. These processes collectively contribute to hyperalgesia. ASC apoptosis-associated speck-like protein containing a CARD, DAMP damage-associated molecular pattern, IL-1 interleukin-1, IL-1R interleukin-1 receptor, IL-18 interleukin-18, MDP muramyl dipeptide, NF-κB nuclear factor-kappa B, NOD2 nucleotide-binding oligomerization domain 2, PAMP pathogen associated molecular pattern, ROS reactive oxygen species, TLR Toll-like receptor, TNF-α tumor necrosis factor-α, TNFR tumor necrosis factor receptor, TXNIP thioredoxin-interacting protein

Inflammasomes are the complexes composed of NOD-like receptors, apoptosis-associated speck-like protein containing CARD and pro-Caspase-1. According to assembled components, they are mainly classified as NLRP1, NLRP3, NLRC4, IPAF, and AIM2. Inflammasomes are recognized as multiprotein signaling platforms orchestrating inflammatory responses and host defense against microbial invasion. Inflammasomes were initially recognized as mediators of danger signals and pathogens.^317^ As the research moves along, their causalities with pain perception, especially inflammatory pain, have been increasingly revealed (Fig. 6).

Nerve injury induces inflammasome activation within several days.^40^ There are significant sex differences in inflammasome activation patterns. In males, high concentration of NLRP1 and NLRP3 inflammasomes can be detected. NLRP3 inflammasome regulates pain perception in both sensory neurons and non-neuronal cells. By contrast, NLRP3 and AIM2 inflammasomes are more abundant in females, with pain signal modulation controlled only by sensory neurons, independent of non-neuronal cells.^318,319^ Activation of inflammasomes promotes the conversion from pro-Caspase-1 to its active form, Caspase-1, which cleaves precursors of IL-1β and IL-18 to produce mature inflammatory factors.^320^ They induce a series of pathological processes associated with pain, including cell death, neuronal excitability, immune overactivation. The brain barrier is subsequently impaired, further aggravating central neuroinflammation.

Most basic studies focus on the upstream regulatory pathways of NLRP3 inflammasome. TXNIP, a critical protein for regulating oxidative stress and inflammatory responses, interacts with NLRP3 inflammasome to promote inflammation activation. TXNIP functions on NLRP3 inflammasome are under strict control of epigenetic modification. For instance, HDAC2 promotes deacetylation of histone H4, decreasing miR-183 expression and antagonizing its inhibitory effects on TXNIP.^321^ TET2 upregulates TXNIP expression at the transcriptional level and facilitates progression of diabetic neuropathic pain.^322^ Activation of TRP family members, TRPV1, TRPA1 and TRPV4, enhances NLRP3 inflammasome activity.^47,323,324^ Moreover, pathological events, like organelle damage and exposure to crystal, also promote NLRP3 inflammasome activity.

The wealth of therapeutic approaches for pain relief are linked to the inhibition of inflammasomes. Inflammasome-related markers are widely employed to reflect the therapeutic efficacy.^325,326^ While research in this field is flourishing, potential side effects of therapies targeting inflammasome activity should be emphasized. The inflammasomes have the important physiological functions. At the early stage of nerve injury, Schwann cells and macrophages rapidly activate inflammasomes, facilitating macrophage phagocytosis of axonal debris. The regulatory factors derived from macrophages can drive Schwann cells to repair damaged nerves.^327,328^ The inflammasome product IL-1β is also critical for the host defense against bacterial invasion.^329^ Therefore, impertinent suppression of inflammasome activity for pain relief may lead to delayed nerve regeneration and immunosuppression, necessitating careful validation and resolution of related adverse events.

### Signaling pathways

Signaling pathways refer to a series of biochemical reactions within cells triggered by specific signals. They collectively regulate nearly all physiological and pathological processes in all aspects of gene expression and functions. The mechanistic associations between pain perception and signaling network abnormality have been gradually uncovered. A large number of preclinical and clinical studies have further demonstrated the potential value of restoration of signaling pathway dysfunctions in alleviating pain. This section will summarize several classical signaling pathways associated with pain aiming to present more integral landscape of molecular mechanisms underlying pain development.

Wnt/β-catenin signaling is one of the most critical pathways, responsible for morphogenesis, carcinogenesis and other physiological processes. Activation of Wnt blocks the formation of β-catenin destruction complex and promotes its transportation to the nucleus, regulating the expression of specific genes. In the animal models with neuropathic pain, Wnt3a, Wnt5a and Wnt10a are significantly upregulated, accompanied with increased nuclear translocation of β-catenin.^330,331^ Some molecular regulators, such as SFRP1, LPAR3, miR-26a-5p and LncCRNDE, jointly control Wnt/β-catenin signaling-mediated neuropathic pain.^331–334^ A recent study demonstrated that GPR177 promotes the secretion of Wnt5a from A-fiber DRG neurons, which further enhances rapid currents of TRPV1+ nociceptive DRG neurons.^335^ This finding has strongly revealed the functions of intercellular communications via Wnt/β-catenin signaling during neuropathic pain progression.

The downstream mechanisms by which Wnt/β-catenin signaling promotes pain hypersensitivity are complicated. Activation of Wnt facilitates trafficking of classical receptors P2X3 and NR2B to the membrane in Ca2+-dependent manners in primary sensory neurons, leading to peripheral sensitization and bone cancer pain progression.^336,337^ VGLUT2 is a critical component of glutamate neurotransmitter system, associated with various kinds of pain. Wnt1 signaling can upregulate VGLUT2 expression and potentiate neuropathic pain development.^338^ Furthermore, Wnt/β-catenin signaling promotes the biosynthesis and release of substance P, TNF-α and IL-18.^339,340^ For the research on pain management, some natural compounds with analgesic properties, like isoquercitrin, persicae semen and resveratrol.^341–343^ Inhibition of Wnt/β-catenin signaling has also shown the potential in treating nociceptive and nociplastic pain.^344,345^ Amazingly, Wnt pathway inhibitors have been applied to treat osteoarthritis-related pain by clinical trials and showed promising performance.^346^

MAPK signaling is another important pathway within eukaryotic signaling networks. There are many branches of MAPK signaling, including ERK, JNK, p38 MAPK and ERK5 pathways. They all participate in the regulation of growth, differentiation, stress and inflammation though with differences in concrete functions. MAPK signaling has been proved as a pivot of TNF-α-mediated pain hypersensitivity. During the progression of acute nociceptive pain, TNF-α activates p38 MAPK and increases the ser110 site of Nav1.7. Hyperphosphorylation of Nav1.7 contributes to its insertion into the somatic membrane of neurons.^347^ The expression of Nav1.8 and Nav1.9 is meanwhile upregulated.^348^ MAPK-ERK-CREB signaling has also been found to increase Nav1.6 expression in the oxaliplatin-induced neuropathic pain model.^349^ Therefore, MAPK signaling has a broad promotion effect on VGSCs, facilitate the formation of pain hypersensitivity. In addition to TNF-α, MAPK signaling is responsible for the pain-inducing effects of other secretory factors. For instance, inhibition of macrophage- and Schwann cell-derived nerve growth factor mitigates neuropathic pain through inhibiting TAK1-MAPK pathway in the CCI model.^350^ It also activates p38 MAPK signaling and upregulates ASIC3 expression in DRG cells, facilitating mechanical allodynia development.^351^ Substance P released from trigeminal ganglion neurons activates satellite glial cells through ERK1/2 and p38 pathways. The hyperactivated glial cells produce more IL-1β and TNF-α and play an accessory role in inflammatory pain.^352^ As stepping into the stage of pain hypersensitivity maintenance, JNK signaling gradually play a leading role in the astrocytes in response to TNF-α.^277^ However, one study showed that glial glutamate transporter can be upregulated by p38 MAPK. This mechanism prevents long-lasting ongoing spontaneous pain. These results suggest that the branches of MAPK signaling may have opposite effects.^353^

There have been various regimens concerning MAPK signaling interference in preclinical research. Antisense oligonucleotides targeting p38 have been synthesized. They effectively inhibit microglia and astrocyte activation through suppressing MAPK signaling, thereby functioning as an analgesic method for inflammatory and neuropathic pain relief.^354,355^ Some clinically applied drugs, such as tetrahydropalmatine, lidocaine and opioids, have been demonstrated to achieve analgesic effects for nociceptive and neuropathic pain, at least partially, through blocking MAPK signaling.^356–358^ A large number of natural compounds may serve as MAPK signaling inhibitors for treating neuropathic pain.^359^

PI3K/Akt/mTOR signaling functions as a regulator of cell survival, proliferation, angiogenesis, metabolism, autophagy, etc. Different with previous introduced pathways, PI3K/Akt/mTOR signaling seems to play a two-sided role in pain development and management. On the one hand, overactivation of PI3K/Akt/mTOR signaling has been detected in models with nociceptive, neuropathic pain and opioid tolerance.^360,361^ Mechanistically, Akt phosphorylates ASIC1a at the Ser25 site, which promotes its forward trafficking and membrane expression.^362^ mTOR activation has been proved to facilitate reconstruction of nociceptive terminals following inflammation, diminishment of ACC synaptic protein involved in neuropathic pain perception. These mechanisms collectively underpin the pain hypersensitivity progression.^360,363^ Furthermore, NALP1 inflammasome activation can be elicited by PI3K/Akt signaling, accelerating the formation of opioid tolerance.^364^ Topical injection or systemic administration of PI3K/Akt/mTOR signaling inhibitors have shown good performance in reversing hyperalgesia and opioid tolerance.^365,366^

On the other hand, however, activation of this signaling may exert analgesic effects. Neurotrophic factor derived from bone marrow mesenchymal stem cells can enhance PI3K/Akt signaling activity, transforming destructive M1 phenotype into regenerative M2 phenotype of microglia. The autophagy is meanwhile enhanced.^367^ These mechanisms restore the abnormal discharging C-fiber neurons.^368^ During the progression of chronic postoperative pain, microglia can downregulate activity of PI3K/Akt signaling in astrocytes, which induces astrocyte transformation into A1 phenotype and promotes the chronicity of pain.^369^ Additionally, PI3K/Akt signaling has been proved to participate in nerve regeneration and alleviate neuropathic pain.^370^ The above evidence has suggested that the functions of PI3K/Akt/mTOR signaling may depend on cell types, pain types and development stages. Crude inhibition or activation of this signaling may be ineffective and bring about potential side effects. It is eagerly required to explore more precise targeted therapies for PI3K/Akt/mTOR signaling interference.

AMPK is a hub regulator of biological energy metabolism. The dysregulation of AMPK signaling contributes to various metabolism-related diseases. AMPK signaling-mediated metabolic disorders have been proved as an important factor of hyperalgesia. For instance, HSP22, a kind of heat shock proteins, is downregulated in the spinal cord neurons of models with nerve injury. Restoration of HSP22 expression improves mitochondrial biogenesis and reduces oxidative stress through activating AMPK/PGC-1α pathway, attenuating neuropathic pain.^371^ Meanwhile, this mechanism underpins osteoarthritis pain relief caused by Sestrin2 overexpression.^372^ AMPK signaling serves as a sensor of intracellular glucose concentrations. AMPK signaling hyperactivity can rapidly reduce TRPA1 membrane expression and its channel activity. High-glucose exposure significantly inhibits AMPK signaling in DRG neurons and potentiates TRPA1-mediated hyperalgesia, which is a critical mechanism underlying painful diabetic neuropathy.^373^ Suppressing NLRP3 inflammasome is another identified mechanism of AMPK-induced analgesia.^374^

AMPK signaling exerts huge impact on non-neuronal cells. Its activation promotes M2-type polarization of microglia and reduced the release of proinflammatory factors.^375^ AMPK signaling has also been found to participate in the functions of endocannabinoid-induced analgesia through reprogramming of the phosphoproteome and bioenergetics of macrophages.^376^ The autophagic flux of Schwann cells is also enhanced by AMPK hyperphosphorylation, attenuating peripheral neuropathic pain.^377^ Taken together, existing studies have shown that AMPK signaling may play a protective role in pain perception. Some drugs and therapeutic regimens as AMPK agonists, such as metformin and caloric restriction diet, have been proved to effectively contribute to pain relief.

In addition to the abovementioned classical signaling pathways, NF-κB, JAK/STAT, TGF-β, Notch, Hippo/YAP1, Hedgehog, cGAS-STING signaling have been proved to be associated with pain modulation. Notably, these signaling pathways have compact crosstalk. One molecular target or pain phenotype may be under control of various pathways. The interlaced signaling network underpins hyperalgesia and pain relief. Likewise, interfering with pivots within signaling pathways may bring about unexpectable side effects, which may contribute to slow paces of clinical translation of agonists or inhibitors targeting signaling pathways. Further studies should strictly inspect their effectiveness and safety.

### Microbiota

The human microbiome, comprising ~10^14^ microbes, is a symbiotic superorganism.^378^ Microbiota reside in the gastrointestinal tract, skin, respiratory tract and reproductive system. The interactions between eukaryotic systems and microorganisms play a significant role in physiological and pathological modulation.^379^ The mechanisms underlying pain perception, particularly gastrointestinal dysfunctions, regulated by microbiota are gradually being uncovered, which can be generally divided into indirect and direct pathways.

For the indirect pathways, microbiota dysbiosis can be recognized by immune systems. Pathogen-associated molecular patterns (PAMPs) refer to the highly conserved structural components of microbiota that are recognized by host cells. PAMPs essentially constitute the molecular immunogenic signatures of pathogens, which include lipopolysaccharide (LPS), peptidoglycan, teichoic acid, and mannose. The recognition of PAMPs contributes to the activation of immune cells, producing proinflammatory cytokines, chemokines and neuropeptides. Their topical and plasma levels are sharply increased, and the crosstalk between immune cells promotes nociceptive pain progression.^277,380–382^ Particularly, the abrupt release of TNF-α caused by microbiota dysbiosis exacerbates nociceptive pain hypersensitivity in the PNS.^277,383^

**Fig. 7:**
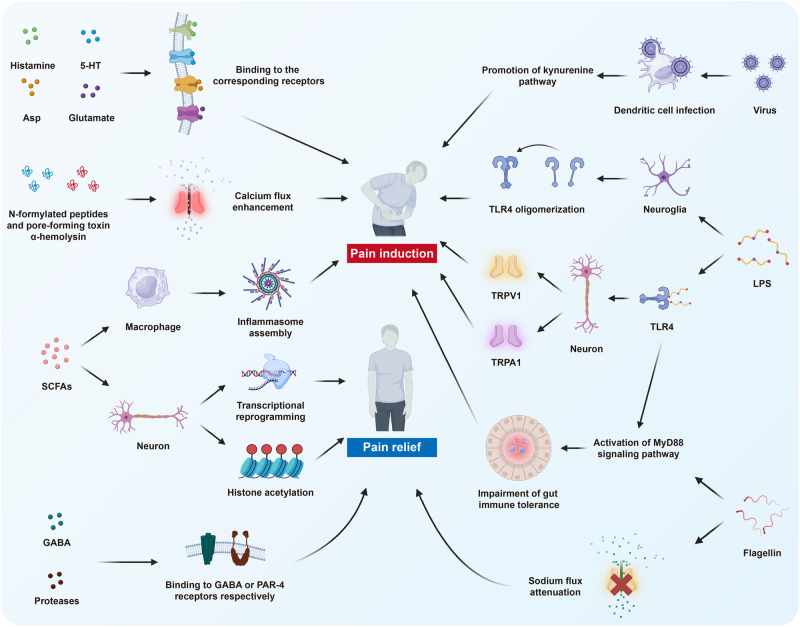
The direct mechanisms underlying regulation of pain perception by microbiota. The microbiota-derived metabolites, including histamine, 5-HT, Asp and glutamate, bind to their corresponding receptors to enhance pain sensation. In contrast, GABA and proteases can respectively bind to GABA and PAR-4 receptors to alleviate pain. N-formylated peptides and the pore-forming toxin α-hemolysin produced by microbiota, enhance the calcium flux in nociceptors and induce pain perception. SCFAs exhibit dual effects. They interact with macrophages, inducing inflammasome assembly and pain perception. On the other hand, SCFAs promote transcriptional reprogramming and histone acetylation in neurons, reducing pain hypersensitivity phenotypes. Viral infection of dendritic cells facilitates the kynurenine pathway, contributing to pain development. LPS possesses the typical properties of pain induction in a TLR4-dependent manner. TLR4 oligomerization in neuroglia, activation of TRPV1 and TRPA1 in neurons and immune tolerance impairment in intestinal epithelial cells through TLR4/MyD88 signaling pathway are mechanisms underlying LPS-induced pain sensation. Another bacterial component, flagellin, likewise attenuates gut immune tolerance through regulating MyD88 signaling pathway. However, it can decrease sodium flux in nociceptors, inversely alleviating pain perception. 5-HT 5-hydroxytryptamine, Asp aspartic acid, SCFA short-chain fatty acid, γ-aminobutyric acid, PAR-4 protease activated receptor, LPS lipopolysaccharide, TLR4 toll-like receptor 4, TRPV1 transient receptor potential vanilloid 1, TRPA1 transient receptor potential ankyrin 1

The direct pathways are more complicated, which are schematically illustrated in Fig. 7. Microbiota-derived compounds are critically involved in the direct pathways. Histamine pertains to one type of amine compound originating from histidine decarboxylation. It plays important roles in perceiving nociceptive information. Gram-positive bacteria utilize pyruvoyl-dependent histidine decarboxylase and gram-negative bacteria employ pyridoxal phosphate-dependent histidine decarboxylase to produce histamine. Gut histamine concentration is correlated with the degree of abdominal pain.^384,385^ A recent study showed that *Klebsiella aerogenes* is a main producer of histamine, and its ectopic histamine metabolism is the culprit of IBS-induced abdominal pain.^386^ Activation of the H1 receptor induces excitation of TRPV1 signaling transduction and sensitization of nociceptive nerves.^387,388^

5-HT is an important metabolite product of gut microbiota, such as *Corynebacterium*, *Streptococcus* and *Enterococcus*.^389^ Plasma 5-HT is significantly increased during the progression of IBS.^65^ A combined network analysis based on IBS patients indicated that visceral hypersensitivity is associated with gut microbiota related to the local 5-HT system.^390^ Long-term exposure to 5-HT enhances its interactions of 5-HT3 and 5-HT4 receptors with cannabinoid signaling, deteriorating the progression of visceral hypersensitivity.^391^ Gut homeostasis modifiers alleviate abdominal pain by restoring normal levels of 5-HT.^392^

Moreover, classical excitatory neurotransmitters glutamate and aspartic acid, together with inhibitory neurotransmitter GABA, are also important products of microbiota and participate in pain modulation. Glutamate and aspartic acid are synthesized by Bacillus bacteria in the guts. The absorbable products facilitate excitability of sensory neurons.^393,394^ Many bacteria produce GABA, including *Lactobacillus*, *Bifidobacterium* and *Bacteroides*.^395,396^ Studies have proved that supplementation with specific gut probiotics effectively increases GABA levels and restores visceral hypersensitivity.^397^ Of note, fungi are also able to produce GABA,^398^ implying the importance of nonbacterial species in pain modulation.

Direct interactions of microbiota with neurons are another direct mechanism of pain induction, independent of immune activation or secreted metabolite regulation. The antigenicity of microbiota constitutive elements endows sensory neurons with cognition capability. Toll-like receptors (TLRs) act as detectors of microbiota invasion, initiating innate immune responses by recognizing PAMPs and activating intracellular signaling pathways. There are 13 functional categories of TLRs, and they monitor different types of PAMPs derived from microbiota.

TLR4 is a critical receptor mediating chronic pain development. Its expression can be detected in the afferent fibers of trigeminal nerves and DRG. Peripheral nerve injury and inflammation trigger significant increases in TLR4 expression in the spinal cord.^399^ LPS is a classical activator of TLR4. TLR4 activated by LPS increases the firing frequencies of TRPV1^+^ neurons, leading to nociceptive, nociplastic and neuropathic pain under bacterial dysbiosis.^380,400,401^ Gut microbiota may interplay with TLR4 receptors and induce oxaliplatin-induced mechanical hyperalgesia.^402,403^ During the progression of IBS, LPS regulates the TLR4-MyD88 receptor signaling pathway to inhibit the synthesis of resolvin D1 in colonic tuft cells, inducing visceral pain hypersensitivity and colonic inflammation.^404^ Apart from peripheral TLR4, upregulation of TLR4 expression in the PFC and hippocampus is observed during abdominal pain progression, providing strong evidence for the crosstalk of the gut-brain axis.^405^

Bacterial flagellin is a typical activator of TLR5. A positive association between TLR5 expression and the abundance of flagellated bacteria has been observed in rats with chronic abdominal pain.^406^ HMGB1 is released by macrophages or necrotic cells under infection or inflammation. HMGB1 acts as a ligand of TLR5 and activates the NF-κB signaling pathway, leading to the development of allodynia.^407^ Nevertheless, one study revealed the analgesic effects of TLR5. Flagellin induces TLR5-dependent blockade of sodium currents of Aβ fibers. The combined use of flagellin and the membrane-impermeable lidocaine derivative QX-314 effectively alleviates mechanical allodynia induced by chemotherapy, nerve injury and diabetic neuropathy.^408^

Several critical but sporadic research studies on direct interactions between microbiota and neurons provide new perspectives on microbiota regulation in pain. LPS can activate TRPV1, TRPM3, and TRPM8 channels located in the DRG.^409,410^ N-formylated peptides and the pore-forming toxin α-hemolysin produced by *Staphylococcus aureus* directly increase calcium flux and action potentials of nociceptors.^25^ The excitability of DRG neurons is downregulated by gastrointestinal commensal bacteria, such as *Faecalibacterium prausnitzii*, through blockade of PAR-4.^411^ Furthermore, acute treatment with the lysate of *Escherichia coli*, rather than purified LPS, dramatically facilitates afferent discharge of colonic mesenteric nerves. Deletion of TLR signaling effects cannot rescue pain hypersensitivity,^412^ implying that there are undiscovered mechanisms and targets by which microbiota directly stimulate nociceptor hyperactivity.

Taken together, the microbiota plays an essential role in pain perception, especially for visceral hypersensitivity. Abnormal microbiota signatures associated with pain have been depicted. The mechanisms underlying bidirectional interactions between microbiota and the nervous system have been gradually revealed, which spawn many future directions for pain diagnosis and treatment.

## Mechanisms of pain sexual dimorphism

**Fig. 8:**
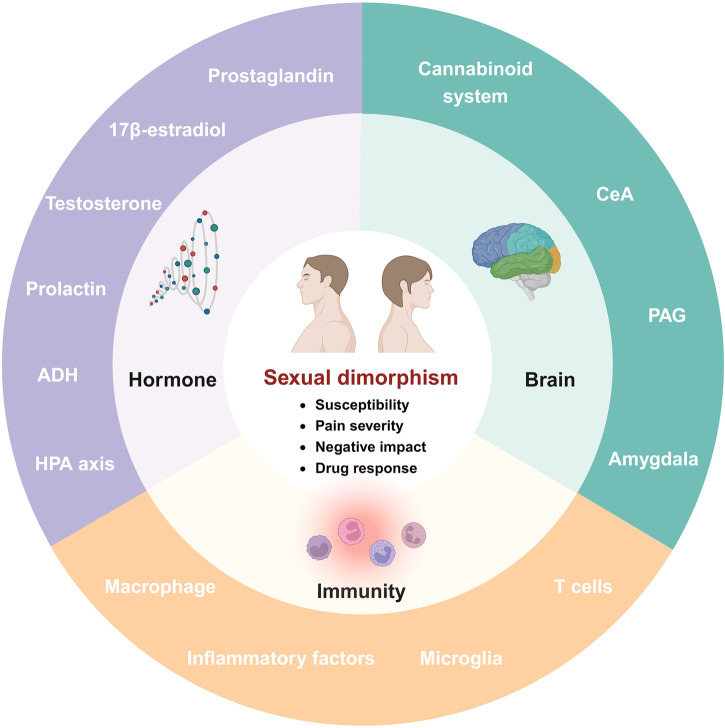
The mechanisms underlying sexual dimorphism of pain. The sexual dimorphism in pain is characterized as four aspects: susceptibility, pain severity, negative impact of pain and responses to analgesic drug. These differences are attributed to variations in hormones, brain function, and immunity. The specific factors in each mechanism, as identified by existing studies, are displayed in the corresponding outer ring. ADH antidiuretic hormone, CeA central amygdala, HPA hypothalamic-pituitary-adrenal, PAG periaqueductal gray

Pain seemingly exhibits a disparity between the sexes. Epidemiological data demonstrate a significant predisposition for pain in females.^413,414^ More basic experiments using female animals, particularly since the beginning of this decade, have advanced our understanding of the characteristics and mechanisms of sexual dimorphism in pain.^415^ This phenomenon is typically characterized by four aspects: higher morbidity susceptibility, stronger pain sensation, more pronounced negative effects and poorer responses to analgesic drugs.^416–418^ Beyond the impact of congenital physiological factors, such as menstruation, the intrinsic mechanisms of pain modulation have been shown to contribute to sexual dimorphism. Significant sex differences in the structures and functions of the CNS and PNS during pain perception have been observed.^419,420^ High-throughput data further reveal global differences in gene polymorphism and expression.^103,421,422^ Some therapeutic regimens have been proposed to address inefficacy of analgesic drugs due to sexual dimorphism.^423^ We will give an overview of the mechanisms by which pain has typical sex preferences, illustrating the sophisticated modulation of pain (Fig. 8).

### Hormone

Sex hormones, crucial determinants of sexual characteristics, affect pain sensitivity through their periodic fluctuation.^424^ Nerve injury, in turn, induces the upregulation of estrogen receptor expression in the DRG, microglia and spinal dorsal horn. 17β-estradiol, a primary component of estrogen, binds to estrogen receptors, regulating interactions between inositol 1,4,5-triphosphate and ryanodine receptors. It subsequently enhances calcium transients and neuronal excitability in response to noxious stimuli. Concurrently, the inflammasome activity and the release of proinflammatory factors are promoted, accompanied with a significant decrease in membrane expression of GABA receptors.^425–427^ Additionally, 17β-estradiol is involved in the functional modulation of specific brain regions, such as the hippocampus, parabrachial and amygdala.^428,429^ Other estrogen metabolites, including 2- and 4-HEMs and 2- or 4-OHE1, directly simulate TRPV1 and TRPA1, contributing to the dramatical reduction in uterine pain threshold.^430^ Conversely, testosterone has been proved to have protective effects against nociceptive pain.^431^

Prolactin is responsible for mammary gland growth and the stimulation and maintenance of lactation. The prolactin receptor (PRLR) in sensory neurons is imperative for initiating pain sensation in females, but unnecessary in males.^427^ The PRLR protein is detectable only in sensory neurons of females, although its mRNA is found in both sexes.^432^ The effects of PRLR on nociceptive pain modulation rely on mutual antagonism between its isoforms PRLR-L and PRLR-S. PRLR-S enhances neuronal excitability, while PRLR-L upregulation selectively rescues pain sensitivity.^433^ Activation of κ-opioid receptor signaling in the hypothalamus induces dysregulation of PRLR isoforms and prolactin production in sensory neurons, accompanied by the release of CGRP.^434^ The neuronal hyperreactivity to stimuli during migraine progression is subsequently enhanced.^435^

Sex differences in the neuroendocrine stress axis are preliminarily revealed. Corticotropin-releasing factor expression in the CeA is elevated in response to stress-induced visceral pain.^436^ Knockdown of β2-adrenergic receptors in nociceptors effectively alleviates hyperalgesia in females, but is inefficacious in males. Blockade of glucocorticoid receptors attenuates pain following paclitaxel-induced peripheral neuropathy only in males.^437^ These findings suggest that sexual dimorphism in pain may be associated with two independent neuroendocrine stress axes. Additionally, prostaglandin and antidiuretic hormone also have close associations with pain susceptivity in females.^438,439^ However, there is still a lack of mechanistic investigations into non-sex hormones.

### Brain

The differences in brain region functions and circuits constitute another important factor in sexual dimorphism in pain. Interestingly, females exhibit more pronounced empathy for pain, which is associated with sex differences in brain region activities.^420^ Most studies focus on the amygdala and PAG. The amygdala, a hub for emotional processing, shows apparent differences in responses and projections between healthy men and women. During the progression of visceral pain, the amygdala in males is more connected with the anterior cingulate subregions and insula, while in females, it shows stronger functional connectivity with prefrontal modulatory regions.^440^ This feature may underlie sexual dimorphism in pain. One study using MRI data indicated that some amygdala subnuclei, including the lateral and basal nuclei in the left hemisphere, exhibit distinct neuronal excitability. These regions act as modulators of the interplay between negative emotions and pain, with changes in their volumes serving as complementary mediators in sexual dimorphism.^441^

The ascending pain transduction tracts are more engaged in preventing pain habituation in males.^442^ The regulatory signals from the CeA project to the ventrolateral PAG (vPAG). Inflammatory pain can induce overactivation of the CeA-vPAG pathway in females, resulting in more severe chronic pain sensations.^443^ In addition to differences in the upstream regulatory pathways, PAG functions also exhibit sexual dimorphism. Critical mediators in the endocannabinoid system, 2-arachidonoylglycerol (2-AG) and anandamide, are inhibited in the PAG of females,^444^ indicating the relative ineffectiveness of endocannabinoid-associated analgesia. Furthermore, fear-conditioned analgesia, which refers to pain relief caused by re-exposure to a condition previously associated with aversive stimuli, is coordinated by the PAG. Males exhibit more robust fear-conditioned analgesia, possibly due to the higher presence of GABA in the PAG of males.^445^ Moreover, congenital structural differences in brain regions, such as thicker posterior insula and precuneus cortices in female migraine patients, may have associations with sexual dimorphism.^446^ The aforementioned mechanisms collectively support the evidence that differences in brain regions contribute to sexual dimorphism in pain sensation.

### Immunity

Males suffering from pain present more pronounced profiles of immune activation, despite females experiencing more intense pain. Among all the immune regulators, microglia have shown the most salient sex-dependent functions. Microglia in males are more sensitive to nociception, as evidenced by increased cell hyperactivity and the release of more pro-inflammatory factors.^447^ Overactivation of microglia leads to higher required doses of opioid analgesia in females.^448^ Peripheral macrophages can also exhibit distinct polarization and motility when stimulated by pro-inflammatory factors in both sexes.^449^ Several key factors have been identified as causes of sexual dimorphism in microglia activity, such as HMGB1, SETD7, and P2X4R. They all activate microglia by enhancing inflammation-associated signaling pathways.^447,450,451^ More importantly, inhibiting microglia activation or antagonizing stimulating factors effectively attenuates hyperalgesia only in males, not in females.^452,453^ These results imply that male pain hypersensitivity is largely dependent on microglia, whereas the link with female hyperalgesia is less clear. In other words, the progress in pain management research through targeting immune responses in males is therefore more promising, particularly with the recent insights into microglia mechanisms. Additionally, dysregulation of proinflammatory factors, including IL-6, IL-23 and TNF-α, is an important reason of poor sensation of nociceptive, neuropathic and nociplastic pain, as well as the drug responses in females.^452,454,455^

In summary, sex differences of hormones, brain regions and immune responses collectively mediate the sexual dimorphism in pain. Nevertheless, this is emphatically not the complete picture, and sexual dimorphism does not simply mean that females are all along at more disadvantaged positions. Women may gain greater adaptation and habituation to persistent heat pain.^456^ Voluntary exercise, like running, is effective in restraining nociceptive stimuli in females by inhibiting inflammatory responses and DRG excitability. Conversely, it enhances the release of proinflammatory factors in males.^457,458^ Furthermore, cognitive disorders following chronic neuropathic pain occur only in males.^459^ Some studies have reported the negative results or even opposite conclusions regarding pain differences,^460,461^ highlighting the complexity of sexual dimorphism in pain modulation. Future research is eagerly required to delve into its deep-seated reasons.

## Mechanisms of pain comorbidities

Pain is not an isolated event. The development of pain sensation is usually predicated on disease progression. Pain, in turn, is likely to cause comorbid physiological disorders, such as depression, anxiety, sleep disturbance and cognitive impairment. These comorbidities greatly reduce the quality of life and increase medical costs. A large number of neural circuits and molecular regulators have been found to promote pain comorbidities. This section will summarize the mechanistic achievements, categorized according to the types of comorbidities.

### Depression

The prevalence of comorbid depression is alarmingly high, approximately ranging from 40% to 60%. There is a significant positive correlation between the severities of pain and depression.^462,463^ The ACC is critically involved in comorbid depression associated with chronic pain. It drives the enhancement in firing and bursting activity of the ACC.^464^ Microglia and glutamatergic neurons are concurrently activated.^465^ The synaptic structures undergo reorganization, facilitating pain-induced depression.^92,466^ The molecular mechanisms underlying these pathological changes are scattered so far. For instance, histone acetylation and phosphorylation of the transcription factor ATF/CREB contribute to the upregulation of MAPK phosphatase-1 expression in the ACC.^467^ Tiam1 coordinates cytoskeleton reorganization and membrane NMDAR stability.^92^ The imbalance of TREM-1 and TREM-2, two receptors expressed on microglia, mediates inflammation outbreaks in the ACC. These molecular events collectively underpin ACC hyperactivity and depressive-like behaviors.^465^

Certain neural circuits, especially those involving the amygdala, play a role in the formation of depression comorbidity. The ACC-amygdala pathway is the core of depression comorbidity in pain. Activation of this pathway in rodents is sufficient to replicate brain transcriptome signatures of clinical patients. The activity of ACC-amygdala pathway is dependent on signal factor Sema4A^468^ The nucleus of the solitary tract and the dorsal raphe nucleus are two brain regions regulating depression. They respond to pain signals and project to CeA.^469,470^ Subsequently, DNMT1 expression in GABAergic neurons is upregulated, partially due to the positive functions of upstream lncRNA XR_351665.^471^ The DNMT1-mediated DNA methylation network suppresses GABAergic neuron activity, inducing comorbid depressive symptoms.^472^ Additionally, some studies reveal the side effects of active protection from neuropathic pain. Neurons of the mPFC and locus coeruleus are activated at the acute phase, which partially block the outburst of pain sensation through enhancing inhibitory signals. It can be regarded as negative feedback for neuropathic pain relief. However, this restorative analgesia induces dysfunctions of the endocannabinoid system in the mPFC and adrenoreceptors in the ACC as pain chronicity develops, resulting in neuropathic pain-induced depression.^473,474^ These findings highlight the complexity of depression comorbidity. It should be noted that single therapy against depression comorbidity may have risks in aggravating hyperalgesia.

The kynurenine pathway accounts for approximately degradation of 95% dietary tryptophan. Tryptophan 2,3-dioxygenase in liver or indoleamine 2,3-dioxygenase (IDO) in other organs is the rate-limiting enzyme of the kynurenine pathway.^475^ Evidence has shown the critical roles of the kynurenine pathway in regulating visceral and inflammatory pain perception.^476,477^ The enhancement in the kynurenine pathway, characterized by an elevated kynurenine/tryptophan ratio, correlates positively with pain sensation.^478^ Kynurenine is then metabolized into a pronociceptive metabolite 3-hydroxykynurenine catalyzed by kynurenine 3-monooxygenase (KMO). After a series of enzymatic reactions in the kynurenine pathway, the final product, quinolinic acid, also participates in neuropathic pain modulation.^479^ For the depression comorbidity, pain induces upregulation of IDO expression both in the hippocampus and liver, accompanied by alterations in metabolites in the kynurenine pathway and increased expression of KMO. Ablation of IDO and KMO functions effectively mitigates depression-like behaviors following chronic pain, providing novel therapeutic targets for attenuating depression comorbidity.

### Anxiety

The core feature of anxiety is excessive worry, manifested as concerns about possible future catastrophes or unhappy events that are disproportionate to reality, or not clearly understood in terms of their objects or contents. Pain dramatically increases incidence rates of anxiety comorbidity.^480^ Some neural circuits and related molecular mechanisms are critically implicated in the development of anxiety comorbidity. The paraventricular thalamic nucleus generates excitatory projections in response to chronic pain. This neuronal input triggers activation of specific neurons expressing nitric oxide synthase (NOS) in the ventromedial PFC. The enhanced synthesis and release of NO promote nitrosylation modification and trafficking.^481^ This molecular event initiates network excitability and anxiety-like behaviors.^482^ The prelimbic cortex (PrL) is a hub of orchestrating nociception and emotion. TNF-α-induced projections from the PrL activate the basolateral amygdala and meanwhile suppress activity of the vPAG, respectively mediating anxiety and neuropathic pain.^483^ Although the vPAG is not involved in anxiety, the front section of PAG can project to the lateral habenula, leading to neuronal hyperactivity in the lateral habenula. This pathway promotes neuropathic pain and anxiety comorbidity.^484^ Moreover, GABAergic neurons in the lateral septum and CART-positive neurons in the bed nucleus of the stria terminalis are activated by chronic pain, projecting to the lateral hypothalamus. These two inhibitory inputs decrease the lateral hypothalamus activity and cause maladaptive anxiety in models with the CFA or SNI-induced model.^485,486^

### Sleep disturbance

Pain-induced insomnia is essentially a defense mechanism under normal conditions. The prominent symptoms are difficulty in falling asleep and frequent arousals. It increases vigilance under potential threats or at vulnerability status for improving survival possibilities of individuals. However, the catastrophic amplification of this action brings significant health burdens for patients with pain. One large-population study has shown nine shared functional connectivity mediating the vicious interactions between sleep disturbance and pain. Notably, the causality from chronic pain to sleep disturbance comorbidity may be stronger than the effects of sleep loss on pain worsening.^487^

The NAc is an important region in regulating reward, addiction and disgust. A specific subset of neurons in the NAc is activated in response to noxious stimuli or upon awakening. This subset exerts inhibitory inputs divergently to the ventral tegmental and preoptic areas. Downregulation of ventral tegmental area activity causes neuropathic pain hypersensitivity, and the projection to the preoptic area leads to sleep disturbance.^488^ The dual functions of the NAc may provide a target for therapies against pain and sleep disturbance comorbidity. Despite the lack of mechanistic research on frequent arousals, an observational study indicated that the arousal infraslow can appear at the interval of 50 s during non-rapid eye movement sleep. Autonomic arousals and local somatosensory sensitivity are significantly enhanced.^489^ This phenomenon underpins the sleep disturbance induced by frequent arousals.

Furthermore, poor sleep, in turn, aggravates pain experience. Long-term sleep disturbance suppresses the functions of the endocannabinoid system in the thalamic reticular nucleus, which promotes projections to the ventroposterior region of the thalamus. This projection is critically involved in hyperalgesia.^490^ Nerve injury causes a significant increase in signal input from the parabrachial nucleus, facilitating the overactivity of cholinergic neurons in the anterior nucleus basalis during non-rapid eye movement sleep. These neurons stimulate pyramidal neurons and hyperalgesia through directly acting on the S1 region.^491^

There are a few investigations on comorbidities of other mental diseases, such as anhedonia, memory impairment and spatial awareness dysfunctions.^136,492,493^ Notably, some neuronal circuits mediate more than one comorbidity of pain. The overlaps between depression and anxiety have attracted considerable attention. Despite distinct clinical symptoms, many brain regions simultaneously regulate depression and anxiety, including the CeA, ACC, locus coeruleus and amygdala.^494–496^ In general, the research on pain comorbidities unveils close associations of pain with other psychiatric disorders. The importance of investigations in this field is not only derived from huge burdens of pain comorbidities. The achievements may provide new diagnostic biomarkers and therapeutic targets. However, the risks of novel therapies in exacerbating mental diseases should not be neglected, given the crosstalk between comorbidities.

## Approaches for pain assessment and diagnosis

Pain, often challenging to describe and quantify in clinical practice, encompasses various factors for assessment and diagnosis, including its site, degree, type, frequency, duration and pathological phenotypes. In the past for a long time, pain assessment has heavily relied on scales, which is highly subjective and lacks universality and accuracy. Misinterpretation of patient-reported pain may cause medication overprescription or poor responses. The emergence of various novel achievements has provided improved options. This section will focus on the methods that have been applied or showing potential for pain assessment and diagnosis.

### Pain questionaries

Pain questionaries are the commonly used tools for pain screening and evaluation, especially for neuropathic pain. The single-dimensional pain scales are the simplest tool, like VAS, FPS-R and NRS. They rely on the single type of visualized information, including numbers, faces and languages. The subjects can finish examination tasks within 1 min. Due to its simplicity and understandability, whereas compromised with accuracy, they are widely used for pain screening. For the multidimensional scales, NPQ, PD-Q, LANSS and DN4 serves as the representative tools. They include more indicators involving psychology, sleep and emotion. Patients suffering from neuropathic pain can be screened out based on symptom description and self-perception. Screening questionnaires enable rapid initial judgments but may compromise assessment accuracy, like NPQ with only 64% sensitivity and 74% specificity in distinguishing neuropathic pain from nociceptive pain.^497^ PD-Q questionnaire overcomes the weakness of yes-or-no options in previous questionnaires and uses quantitative scoring options instead. It considerably improves the evaluation accuracy.^498^ Until now, PD-Q has been widely employed in screening of chronic neuropathic pain.^499^

Evaluation questionnaires are designed to assess the patient’s symptoms and evaluate the effectiveness of clinical therapy. NPSI, is a self-administered questionnaire developed to describe the clinical symptoms of pain. Unlike the screening questionnaire, NPSI focuses on assessing the effectiveness of pain management and delineation of pain subtypes.^500^ BPI, with advantages of rapid test and wide-range application, is mainly used to evaluate the pain severity and the impact on quality of life. It has been proved as a reliable evaluation tool for diabetic neuropathy pain.^501^ OASIS is a questionnaire used to assess different pain dimensions in osteoarthritis patients with OA. Its most obvious feature is the assessing changes in pain characteristics over time, suggesting that it could be used in the future for drug studies to assess patient responsiveness to treatment.^502^

As a rapid evaluation method of pain, questionnaires have prominent characteristics. They are able to offer efficient and cost-effective pain assessment, promoting accessibility for patients and interpretability for doctors. The subjective sensations are the most important indicators of pain, since pain severity determines the clinical outcomes of individuals. However, pain questionnaires cannot be used for diagnosis and not encouraged to serve as the sole evaluation tool. More methods should be conducted alongside to consolidate the clinical results.

### Quantitative sensory testing (QST) and neurophysiological tests

QST is a psychophysical detection method to assess the perception of touch, vibration, proprioception and sensitivity to various stimuli like pinprick, blunt pressure and temperature in experimental settings. It is commonly applied in mechanistic investigations for providing personalized medicine. As an important neuroelectrophysiological technique, QST is valued for simple operation, non-invasive nature and robust repeatability.^503^ It stimulates different fibers to produce sensations. Temperature sensation is used to examine the functions of Aδ and C fibers, while vibration sensation serves as a marker of Aβ nerve fiber activity.^504,505^ QST typically categorizes pain disorders into sensory loss, thermal hyperalgesia, and mechanical hyperalgesia. Quantitative thermal testing (QTT), a kind of QST, is the most widely used sensory testing. QTT contains four main indicators, including cold detection, warm detection, cold pain threshold and heat pain threshold. They collectively reflect the nerve fiber integrity, especially small fibers.^506,507^ QTT is suitable for patients with lumbar disc herniation who have inconsistent imaging and clinical manifestations or contraindications to MRI examination.^508^ It also shows effectiveness in trigeminal neuralgia-associated pain assessment according to alterations in temperature perception thresholds.^509^

Although QTT effectively compensates for the questionnaire shortcomings, it still has obvious limitations. As a method causing additional pain experience and psychological stress, QTT is commonly applied in experimental research, far from clinical use. Similar to other psychophysical testing methods, QTT requires active engagement of patients, and test results are susceptible to the psychological state.^510^ The lengthy examination can cause fatigue and boredom, affecting the accuracy of pain assessment.^511^ Administration of analgesic drugs, like opioids, may also interfere with detection results.^512^ Therefore, the potential of QTT in pain evaluation needs more in-depth exploration to figure out its optimal application conditions and reference value.

Unlike QST, which is used to evaluate small-fiber neuropathy, neurophysiological tests are primarily used to evaluate large noninvasive afferent fibers. These tests include nerve conduction, trigeminal reflexes (including the blinking reflex), and somatosensory evoked potentials.^513^ Some tests achieved 94% specificity and 87% sensitivity in diagnosing certain neuropathologic pain.^514^ A comprehensive clinical examination, pain questionnaire, QST and neurophysiological tests are helpful for the clinical assessment of patients with neuropathic pain.

### Brain imaging

The activities of specific brain regions are associated with pain severity. Based on these findings, brain imaging techniques have been developed, including structural MRI (sMRI), functional MRI (fMRI) and electroencephalograph (EEG). Brain imaging has emerged as a potential diagnostic biomarker for each type of pain. fMRI is an imaging technique developed on the basis of traditional MRI. It analyzes functional brain network connectivity, detecting neural activity through blood oxygen level-dependent signals. High signal intensities in fMRI are correlated with increased pain perception processing in specific brain regions. The data derived from fMRI can distinguish patients with acute or chronic nociplastic pain from healthy controls with over 63% accuracy.^515,516^ sMRI, engine by AI, utilizes gray matter images for diagnosis, achieving over 70% accuracy based on machine learning models.^517,518^ A prediction model constructed by squares-discriminant analysis likewise accurately distinguishes patients with chronic abdominal pain from the healthy controls.^518^ EEG is employed to detect brain oscillatory activities and functional connectivity changes at different frequencies. It has presented good performances in pain diagnosis due to its properties of ease of use and cost-effectiveness.^519,520^ More importantly, EEG serves as a cross-site pain diagnostic biomarker to differentiate patients with different types and severities of pain.^521^

However, constrained by the limitations of cross-sectional studies, these investigations have not delved into other pain biomarkers like monitoring and prognostic markers. This underscores the need for longitudinal studies focused on identifying dynamic detection and prognostic biomarkers. Long-term observation of subacute patients has shown that changes in NAc volume often correlate with the transition to chronic pain,^522,523^ indicating the potential of brain imaging as a prognostic tool. fMRI in identifying prognostic biomarkers for pain has been utilized.^524^ The functional connectivity between the NAc and PFC, which strengthens with persistent pain, can predict chronic back pain with an area under the ROC curve of 0.81.^525^ This connectivity’s effectiveness in predicting short-term pain reduction in chronic pelvic pain syndrome has been validated with 73.1% accuracy.^526^

Despite the potential of these models in pain prognosis prediction, generalizing them to all pain types is challenging due to sample size limitations and the practicality of longitudinal studies. Recently, several pain-focused databases like OpenfMRI, OpenPain, and the Pain and Interoception Imaging Network repository have been developed, which should facilitate the construction of larger-scale, more reliable prediction models.

In summary, brain imaging’s non-invasive nature and reproducibility make it an objective tool for pain mechanism research, drug action site identification, and intervention therapy development. However, there is a current shortage of longitudinal brain imaging biomarker studies. Future research should focus on large-scale studies utilizing shared data to develop composite biomarkers. Most importantly, individualized pain diagnosis and treatment based on patient classification is a critical future research direction.

### Molecular biomarkers

Given the close correlations, associations, and causal links between pain and molecular mechanisms, certain key molecules hold potential in clinical diagnosis (Table 2). This research area is an increasingly mainstream. The genetic variants have been linked to neuropathic pain, such as *Hla*, *Comt*, *Oprm1*, and *Gch1*.^527^ Large cohort studies have made initial forays. An investigation using the data from Genome Wide Association Study identified 171 differentiated mutant phenotypes in neuropathic ocular pain.^206^ Many targets, however, lack universality due to varied mutation sites.^528,529^ In the future, machine-learning algorithms may enable the combined application of multiple genetic variants.

**Table 2:** The summary of molecular biomarkers in pain diagnosis

Biomarker	Sample	Disease	Number of participants	AUC/Accuracy	Sensitivity	Specificity	Performance	Ref.
OPRM1, OPRK1, OPRD1, SIGMAR1	Serum	Chronic pain	58	0.806	0.937	0.674	The panel effectively distinguishes patients requiring different doses of opioids for analgesia.	^753^
sICAM-1	Serum	CLBP	21	0.890	0.830	0.760	sICAM-1 can be used to evaluate CLBP patients with different pain intensities.	^754^
DNAJC18, CNTN1	Serum	Psychiatric patients with pain	213	0.780/0.630	-	-	DNAJC18 and CNTN1 can predict pain severity in men and women, respectively.	^755^
F13B	Serum	Postoperative pain	80	0.859	-	-	Preoperative serum F13B predicts the required postoperative dosages of sufentanil for gastric cancer patients.	^756^
Gal-9	Serum	Endometriosis	135	0.973	0.940	0.938	Gal-9 effectively differentiates endometriosis patients from healthy controls.	^757^
GTF2H2, KLHL5, LRRC37A4P, PRR24, MRPL23	Skin	Neuropathic pain	227	0.830	-	-	This gene signature can differentiate neuropathic pain patients from healthy controls.	^530^
MYC, STAT1, TLR4, CASP5WLS	Serum	Chronic neuropathic pain	93	0.852	-	-	The combination of MYC, STAT1, TLR4, CASP5, and WLS expression serves as a biomarker panel for distinguishing chronic neuropathic pain patients from healthy controls.	^758^
IL-6	Serum	UAP	1038	0.800	-	-	Serum IL-6 level combined with clinical imaging and patient subjective feeling can effectively differentiate UAP patients from healthy individuals.	^532^
Serum IL-4, peritoneal fluid IL-2, IL-4	Serum and peritoneal fluid	Endometriosis	50	0.660/0.710/0.700	0.636/0.727/0.697	0.647/0.706/0.588	Serum IL-4, peritoneal IL-2 and IL-4 are able to differentiate endometriosis patients from controls.	^533^
TNF-α	Serum	Neuropathic pain	140	0.840	0.736	0.794	Serum TNF-α level can predict the progression of neuropathy in patients with spinal cord injury.	^531^
sPIICP	Serum	Osteoarthritis	212	0.980	0.962	0.921	sPIICP is used to evaluate the severity of osteoarthritis patients.	^759^
OPG	Serum	CRPS	54	0.800	0.740	0.790	OPG, as a serum marker, can effectively distinguish CRPS patients from healthy controls.	^760^
11 metabolites (e.g., methylmalonic acid, xanthurenic acid)	Urinary	Chronic pain	487	0.749	-	-	A FPI model based on 11 metabolites effectively differentiates chronic pain patients from healthy controls.	^535^
ATP	Urinary	IC/BPS	106	0.811	-	-	Urinary ATP has predictive value in the diagnosis and evaluation of IC/BPS severity.	^761^
Vitamin D	Serum	Rheumatoid arthritis	93	0.710	0.484	0.855	Vitamin D shows good predictive value for the occurrence of neuropathic pain in patients with rheumatoid arthritis.	^762^
NLR	Serum	IC/BPS	240	0.765	0.760	0.643	Serum NLR differentiates patients with IC/BPS from healthy controls.	^763^

*AUC* area under curve, *CLBP* chronic low back pain, *CRPS* complex regional pain syndrome, *FPI* foundation pain index, *IC/BPS* interstitial cystitis/bladder pain syndrome, *NLR* neutrophil-to-lymphocyte ratio, *UAP* urgent abdominal pain

Besides gene mutations, gene expression products exhibit more promising potential in pain diagnosis, though lacking direct and robust evidence. The transcriptome analysis has shown that GTF2H2, KLHL5, LRRC37A4P, PRR24 and MRPL23 are highly expressed in the serum of patients with neuropathic pain. The diagnostic model constructed by this gene expression signature can reach an area under curve (AUC) of 0.83.^530^ Combinational use of anti-inflammatory and pro-inflammatory cytokines in serum, such as IL-4, IL-6, and TNF-α, has been proved as potential biomarkers of neuropathic pain or visceral hypersensitivity, with AUC at least 0.66.^531–533^ Metabolomics has led to the emergence of serum metabolites as pain biomarkers. Patients with chronic pain often have abnormal serum levels of kynurenine pathway metabolites,^534^ although their diagnostic efficacy remains unvalidated. Urine-based models using multiple metabolite indicators provide better predictive value, with an AUC of 0.749.^535^

Molecular probes combined with imaging are increasingly being used for diseases requiring biopsy, like tumors.^536^ They can significantly enhance diagnostic efficiency, particularly for small lesions.^537^ This progress can provide enlightenment for pain diagnosis. The molecular probes can be designed to interact with pain biomarkers. Magnetic Resonance Spectroscopy, a new MRI technology, could potentially detect molecular pain biomarkers, improving diagnosis and continuous condition monitoring. Signal intensity changes could also monitor drug effects, aiding in dynamic treatment adjustment. Importantly, molecular probes have potential in informing individualized intervention regimens.

The other techniques like skin biopsy for peripheral neuropathy and gut microbiome analysis for pain severity correlation offer potential diagnostic avenues.^538,539^ Skin biopsy is an examination method to investigate nociceptive fibers in the human epidermis and dermis. It is used to investigate peripheral neuropathy, including diabetic neuropathy and infectious neuropathy, with sensitivity and specificity approaching 90%.^540^ The primary advantage of nerve ultrasound lies in its non-invasive and convenient nature, enabling the assessment of peripheral neuropathy through the use of a high-resolution ultrasound probe, as opposed to skin biopsy.^541^ However, the positive findings of both skin biopsy and neuroultrasound cannot be directly linked to the diagnosis of pain.^542^ The primary challenge in pain diagnosis is the absence of objective standards and effective assessment approaches. The progress in pain diagnosis seriously lags behind the research on pain mechanisms and therapies. Integrating objective diagnosis with psychosocial research to develop comprehensive clinical diagnostic approaches remains a complex task requiring further investigation and validation.

## Intervention methods for pain relief

**Fig. 9:**
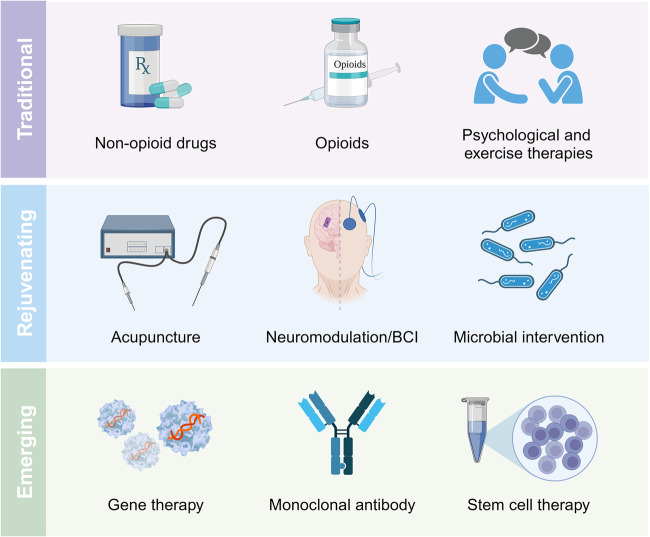
The summary of currently developed therapies for pain management. The nine methods are categorized into three groups, including traditional, rejuvenating, and emerging therapies. BCI brain-computer interface

Human has been grappling with pain throughout the long history. The complexity of pain’s etiologies, including biological, psychological and social factors, necessitates multidisciplinary treatment approaches. Primary disease therapies are crucial. However, for the incurable diseases and pain chronicity, specialized analgesic methods are meanwhile required. The surge in preclinical research contributes to the emergence of many novel therapies and intervention targets. Identification of underlying mechanisms provides additional insights into the efficacy of widely used therapies. The multimodal approach mainly includes three aspects according to the development stages: the traditional, rejuvenating and emerging therapies (Fig. 9). The traditional therapies refer to the widely applied approaches with mostly identified mechanisms underlying pain relief. By contrast, although the rejuvenating therapies have been used for pain management for a long history, their mechanisms are largely unknown and gradually being revealed. Some novel strategies hail from the old and plain therapies by virtue of modern biotechnology. The emerging therapies are completely the products of biotechnological advances, which require further explorations and validations. This section aims to summarize current achievements, offering a clear and comprehensive overview for researchers and clinicians.

### Non-opioid pharmaceuticals

Non-opioid drugs are the most commonly used for pain management by virtue of their high dependability and safety. They mainly include NSAIDs, antidepressants, gabapentinoids, antiepileptics and cannabinoids. The non-opioid pharmaceuticals are applicable to different types of pain. For example, NSAIDs are evaluated as the first-line treatment for inflammatory pain, while antidepressants and antiepileptics are preferred for neuropathic pain caused by nerve injury and neuropathy.

NSAIDs function as cyclooxygenase (COX) inhibitors, reducing prostaglandin production. COX-2, inducibly expressed in immune cells under specific stress events like infection and inflammation, is targeted by NSAIDs to suppress immune cell activation and encourage oligodendrocyte differentiation.^543^ This leads to a decrease in proinflammatory factors and restoration of nociceptor sensitivity. In addition to the peripheral effects, NSAIDs also impact activities of brain regions, such as the PAG, CeA and spinal cord,^544,545^ which serve as important central mechanisms of analgesic effects of NSAIDs. They have been recommended as first-line or primary analgesic drugs by many authoritative guidelines, such as PROSPECT for postoperative pain, ACP/AAFP guideline for musculoskeletal acute pain, ESCEO/OARSI guidelines for osteoarthritis and ACP guideline for gout.^546–549^

Tricyclic antidepressants (TCAs) inhibit the reuptake of norepinephrine and 5-HT, thereby increasing monoamine neurotransmitter concentrations in synaptic clefts. According to the evidence of basic research, 5-HT enhances the inhibitory effects of serotoninergic system on the descending spinal bulb, together with the influences on β2-adrenergic receptor-mediated antiallodynic action.^550^ The depression-independent mechanisms have also been identified. TCAs modulate opioid and endocannabinoid systems.^551,552^ The densities of membrane ion channels and glutamate transporters are downregulated.^553,554^ They likewise exhibit the anti-inflammatory properties.^555^ TCAs are recommended as the first-choice medications for neuropathic pain according to the IASP guideline.^556^

Selective serotonin reuptake inhibitors (SSRIs), such as fluoxetine, paroxetine and sertraline, are another kind of antidepressants. They have pharmacological properties of inhibiting 5-HT reuptake, increasing synaptic 5-HT concentrations. The regulatory effects of 5-HT depend on subtypes of 5-HT receptors, which have various and even opposite functions on pain modulation. Overall, SSRIs act as pain suppressors, but a literature review of related clinical trials has shown that the efficacy of SSRIs is less effective than that of TCAs, possibly due to canceling various receptors out.^557^ Their wide-range pharmacological actions further increases risks of side effects. SSRIs are not used as the first-line pharmaceuticals in clinical pain management.

Serotonin-norepinephrine reuptake inhibitors, like venlafaxine and duloxetine, show better performance in treating neuropathic pain because of the additional effects on noradrenaline. It serves as the first-line medication for neuropathic pain recommended by the IASP guideline.^556^ A multicenter clinical trial called OPTION-DM investigated the efficacy of sequential administration supplemented with pregabalin. This regimen effectively alleviated diabetic peripheral neuropathic pain but with a significant increase in nausea compared to monotherapy.^558^

Ketamine is known for rapid-acting antidepressant properties. Preclinical studies have shown that its analgesic effects are mostly dependent on NMDAR antagonism, blocking the wind-up phenomenon in the spinal dorsal horn.^559^ The ascending inhibitory pathway activity and antiinflammation are also promoted. The ketamine metabolite, 2R, 6R‑hydroxynorketamine, activates mTOR signaling in PFC, which in turn enhances ketamine efficacy.^560^ This metabolite also downregulates CGRP release through inhibiting the activities of TRPV1 and TRPA1.^561^ For clinical practice, ketamine is recommended to treat moderate-severe postoperative pain by the ASRA/AAPM/ASA consensus guideline. Patients with opioid tolerance or obstructive sleep apnea may gain benefits from ketamine for pain relief due to its properties of compensate opioid efficacy and decreasing opioid requirements.^562^

Gabapentinoids are the classical antiepileptics. Basic studies have shown that gabapentinoids bind to α2δ subunit, decreasing ion influx through neuronal VGCCs. Local administration of gabapentinoids inhibits responses to evoked stimuli.^563^ The effects of gabapentinoids partially rely on the opioid system, involving the rostral ACC and downstream mesolimbic reward circuits engaged in learned pain-motivated behaviors.^564^ Gabapentinoids have been recommended for painful diabetic polyneuropathy.^565^ Pregabalin has better efficacy for the treatment of post-total joint arthroplasty than gabapentin.^566^ However, gabapentinoids are not or weakly recommended for other types of postoperative pain according to the PROSPECT guidelines because of their high risk-benefit ratio.^567,568^ Ongoing clinical trials are trying to expand indications of gabapentinoids in pain management.^558,569^

The molecular mechanisms of cannabinoids are complicated, dependent on cannabinoid receptor types, including cannabinoid receptor-1 (CB1) and cannabinoid receptor-2 (CB2). CB1 activation in the mPFC inhibits activities of dorsal horn and peripheral neurons, preventing neuropathic pain chronicity.^570^ The circuit from the zona incerta to posterior complex of the thalamus is critically involved in pain sensation. The hyperactivity of CB1 specifically expressed in axon terminals of this circuit mitigates nocifensive responses via presynaptic inhibition.^571^ Intriguingly, CB1 counterintuitively sensitizes TRPV1 channel during prostaglandin E2-induced inflammation. The overactivation of TRPV1 inhibits action potential through dampening the depolarization rate in the capsaicin-induced model.^572^

CB2, enriched in immune cells, are linked to inflammation. CB2 activation decreases HDAC1 and IKBα expression, as well as promoting the release of anti-inflammatory cytokine IL-10 in microglia.^573^ The levels of proinflammatory factors are declined, such as IL-1β and TNF-α, accompanied by NLRP3 inflammasome deactivation.^574,575^ CB2 effects are not limited to peripheral inflammation inhibition. 2-AG can bind to CB2 receptor in the ACC and promotes fear-conditioned analgesia.^576^ Additionally, CB1 and CB2 receptors have overlaps in mechanisms, like interactions with Nav1.7 and Nav1.8 to inhibit neuronal activity.^577,578^ However, the endocannabinoid system can activate GABAergic neurons in the mPFC. The excessive release of GABA may increase risks in depression comorbidity.^473^ Researchers should be cautious in cannabinoids-induced negative emotion, particularly given the increasing trend of healthy individuals overusing commercial cannabinoids, like cannabidiol (CBD) gummies or water.

A large number of clinical trials have explored the efficacy and appropriate administration regimens of cannabinoids. For instance, δ-9-tetrahydrocannabinol (THC) alleviates chronic neuropathic pain compared to placebo therapies. The reduction in functional connectivity between the ACC and sensorimotor cortex is observed, suggesting that cognitive-emotional modulation is a critical mechanism underlying its analgesic effects of THC.^579^ A meta-analysis including 16 clinical trials and 1750 cases showed that cannabis-based medicines are significantly effective in chronic neuropathic pain relief.^580^ Furthermore, CBD can exert profound pharmacological interactions with THC.^581^ A phase-II adaptive Bayesian trial indicated that CBD can restore cannabis use disorder.^582^ However, many studies have reported the negative results of cannabinoids, involving acute nociceptive pain, abdominal pain, postoperative pain and inflammation-associated pain.^583–586^ A meta-analysis focusing showed that existing data do not support cannabinoids as an effective drug for both chronic non-cancer and cancer related pain.^587,588^ The effects of medical cannabinoids differ. Only dronabinol and nabiximol exhibit significant therapeutic effects on pain associated with a variety of diseases.^589^ The transient adverse side effects further impaired application value of cannabinoids.^587^ Taken together, cannabinoids are suitable for the treatment of neuropathic pain, rather than other kinds of pain. The drug types, administration dosage and regimens need to be extensively investigated by subsequent clinical trials.

There are several challenges of clinical trials on cannabinoids. Most current studies evaluated pain intensities based on single pain indicators. However, quality of life, sleep, motivational and cognitive dimensions are also have closely associated with patient pain experience. Studies should include more comprehensive indexes to assess analgesic effects of cannabinoids. The interactions with other drugs, like opioids and NSAIDs, are still lacking. The efficacy and potential risks of combinational use should be carefully clarified. The investigations into optimal therapeutic ratios of cannabinoids for different pain conditions are also required, which may provide proofs for developing strategies of separating analgesia from adverse effects. Furthermore, real-world data concerning cannabinoids are currently absent. The data can better illuminate benefits and harms of cannabinoids.

### Opioids

Opioids are analgesic drugs usually used for treating many types of moderate and severe pain at all ages, based on extensive evidence-based medicine. They play a vital role in clinical symptomatic and palliative therapies. Emerging clinical trials have explored novel opioid regimens. For instance, an international, open-label trial gives a strong recommendation for two-step cancer pain management, which refers to bypassing weak opioids in the pathway from non-opioid therapies to strong opioids. This regimen can decrease the cost of two-step approach and achieve the comparable efficacy.^590^ The combinational use of CBD effectively improves patients’ quality of life receiving opioid treatment.^591,592^ The sustained-release and topically administered forms have entered clinical trials for improving analgesic efficacy and reducing adverse event risks.^593,594^

Opioids are agonists of μ, κ, and δ receptors, with diminishing effects across these subtypes. Opioid-induced analgesia involves multiple mechanisms. The canonical manner is the suppression of adenylyl cyclase and high-threshold VGCCs through G protein coupling pathways. The inwardly rectifying potassium channels are meanwhile activated, accompanied with inhibition of TRP family members, VGSCs and ASICs.^595–598^ These events jointly decrease neuronal excitability and excitatory neurotransmitter levels. In the brain’s reward circuits, opioids mitigate GABA-driven inhibitory neurotransmission. The suppression on dopaminergic neurons in the striatum and PFC is reversed.^599,600^ The proinflammatory neuropeptide release is also downregulated, further promoting the analgesic effects of opioids.^601^

The side effects of opioids are the vital reasons for a series of clinical and social problems, mainly including tolerance, hyperalgesia, respiratory depression and gastrointestinal reaction. They are driven by complicated mechanisms, but some crucial cross nodes have been identified. β-arrestin 2 is a negative regulator of GPCR signaling, implicated in opioid tolerance, addiction and respiratory depression through coupling with intracellular and cytoplasmic regions of phosphorylated μ receptors.^602^ Biased agonists with reduced β-arrestin 2 recruitment, like 2S-LP2 and EM-2, are being developed to alleviate these effects.^603,604^ However, there are dissenting opinions that β-arrestin 2 as a scaffolding protein is unlikely to be an ideal pharmacological target. Evidences have shown that severe side effects are not observed in mice with ablation of β-arrestin 2 functions,^605,606^ implying that unknown mechanisms independent of β-arrestin 2 may contribute to chronic opioid tolerance.^607^ Instead, targeting recruited molecules of β-arrestin 2, like vasopressin 1b receptor, may be a promising approach.^608^

Neuroglia cells are involved in opioid tolerance and hyperalgesia. MAPK/NF-κB signaling activation in microglia promotes release of proinflammatory factors and upregulates expression of TLR4.^609^ Inhibitors of MAPK/NF-κB signaling can attenuate opioid-associated side effects in rodent models.^610–612^ CR4056, an imidazoline I2 receptor ligand, suppresses microglia activation and enhances analgesic effects of morphine.^613^ NMDAR in astrocytes is another potent target. The inhibitors targeting NMDAR effectively block intercellular communications between astrocytes and neurons, alleviating opioid tolerance.^614^ Antagonizing IL-33-mediated crosstalk between astrocytes and oligodendrocytes also prolongs morphine’s analgesic effects.^615^ Therefore, neuroglia may serve as promising targets in mitigating opioid side effects.

Tramadol is a weak opioid agonist widely used for pain relief. Tramadol is primarily used for postoperative pain and chronic musculoskeletal pain management. It has also been recommended as the non-first-line drug for the treatment of neuropathic pain by CPS and EFNS guidelines.^616,617^ Additionally, it can be safely and effectively used for delivery analgesia without affecting the newborn’s respiration. Recently, a chewable tablet has been invented and used for children, further proving its safety.^618^ For the basic mechanistic research, tramadol exhibits a dual mechanism, primarily through the activation of opioid receptors in the CNS presynaptic membrane and the inhibition of 5-HT and norepinephrine reuptake in the presynaptic membrane of the descending inhibitory system of spinal cord. This dual mechanism allows tramadol to achieve analgesic intensity comparable to opioids at appropriate dosages.

Otherwise, nitric oxide, a gasotransmitter, activates presynaptic and postsynaptic guanylate cyclase, leading to the production of cGMP. This signaling further promotes opioid tolerance proved by basic studies. NOS inhibitors, like L-NAME and aminoguanidine, attenuate morphine tolerance. Repeated administration can further alleviate the withdrawal symptoms of opioids.^619,620^ Despite promising preclinical results, the translational speed has slowed markedly in recent years. The clinical application of NOS inhibitors remains cautious, pending further evidence.

### Psychological and exercise therapies

Restoration of psychological and behavioral disorders has demonstrated significant value in pain management. CBT, which integrates behavior modification with psychotherapy, is a gold-standard approach for treating mental diseases. Its analgesic effects are applicable to adults at all ages with chronic pain, such as chronic low back pain, osteoarthritis and IBS.^621,622^ Notably, a recent meta-analysis based on 153 trials and 8713 participants has strongly recommended CBT for management of chronic pain associated with temporomandibular disorders.^623^ To further promote application of CBT, clinical trials began to investigate the efficacy of online CBT. The results showed its comparable competences with traditional psychotherapies at the dramatically lower cost,^624,625^ providing new directions of CBT development.

For the mechanistic investigations, CBT induces global alterations in brain region activities. Prevention of pain catastrophizing is an important mechanism of CBT, which relies on the regulation of the ventral posterior cingulate cortex, a hub of the DMN. CBT impairs the connectivity between the somatomotor and salience network regions in fibromyalgia patients.^626^ The connectivity strength involving the ventral posterior cingulate cortex is negatively correlated with CBT efficacy.^627^ The mPFC is another key node in the DMN. CBT facilitates new long-term potentiation connections in the mPFC with other critical regions, like the amygdala and insula, underpinning significant correction of chronic nociplastic pain.^628,629^ The enhanced crosstalk of the amygdala with the ACC, frontal and precentral gyrus is also related to the responsiveness to CBT.^630^ Although most evidences only demonstrate its correlations with brain region activities, these extensive and profound influences suggest the substantial potency of CBT.

Exercise is another cost-effective therapy against pain, which has been strongly recommended by recent guidelines for the clinical management of pain associated with motor system, including fibromyalgia, osteoarthritis, low back pain, chronic musculoskeletal pain and temporomandibular disorders.^549,623,631–633^ It has also been evaluated as an important component of therapies against cancer and neuropathic pain.^634,635^ Inflammation mitigation is a major mechanism of exercise, which is mentioned above in the section of pain sexual dimorphism.^457,458^ The descending regulatory pathway is an important target of exercise. Regular exercise increases the concentrations of endogenous opioids (β-endorphin and enkephalin) in the PAG and RVM.^636^ The activities of regions, such as the anterior insula, left dorsolateral PFC, locus coeruleus and midbrain reticular formation, are globally altered in patients with nociplastic pain.^637,638^ However, due to various methods and intensities of exercise therapy, the optimal prescription and delivery for specific diseases should be extensively discussed based on more high-level basic and clinical research.

Placebo effect refers to the phenomenon that symptoms are alleviated through psychological functions produced by patients’ belief after receiving dummy treatment. It is especially common in the pain research. The forms of placebos are various, such as tablets, pseudostimulus and sham operation. Their efficacy rivals some classical modalities.^639^ Recent investigations using virtual reality technology have proved that physical entities are unnecessary for pain management.^640^ The adjuvant functions in synergistically enhancing other therapies have also been demonstrated.^641^ On the other hand, the reliability of existing analgesic methods is questioned because it is hard to tell whether placebo effect is involved. It is great pleasure to see that project designers of clinical trials have recently been aware of this confounding factor, leading to recalibration of specific therapy effectiveness.

Activation of opioid and endocannabinoid systems is the dominant mechanism of placebo-induced analgesia, which hints that placebos may become alternative therapies of opioids and cannabinoids.^642^ The PFC, insula and somatosensory cortex have been found to be engaged in this top-down effect, together with processing pain anticipation and perception by the thalamus and brainstem.^643,644^ Some researchers hold the view that functional connectivity may be more sensitive for manifesting placebo effect than isolated brain regions. The decreased connection between the left medial PFC and bilateral insula, responsible for cognition modulation, is correlated with placebo effect in patients with chronic back pain.^645^ The circuits from prefrontal cognitive to pain processing regions also serve as indicators of responsiveness to placebos.^644^ Furthermore, nocebo has properties of hyperalgesia induction. Existing basic studies have revealed totally different neural networks subserving placebo and nocebo effects,^646^ further validating the complexity of pain perception.

In addition to the above methods, mindfulness, short-term dynamic psychotherapy and hypnosis are other psychotherapies for pain management. The research on psychological and exercise therapies has thrived. However, a disparity exists between the abundance of clinical trials and the scarcity of basic research, partly due to challenges in replicating psychological or voluntary exercise in animal models. Future studies should address this imbalance and develop more foundational experimental methods.

### Acupuncture

Acupuncture originates in ancient China and its effectiveness has been confirmed through clinical practice. According to the guidelines, acupuncture can serve as an alternative and complementary therapy for pain management, especially for cancer, low back pain and postoperative and osteoarthritis pain.^546,632,647,648^ Additionally, clinical trials stimulation of pain-specific acupoints produces inhibitory effects of comorbidities of depression, anxiety and sleep disturbance.^649,650^ The underlying principles of acupuncture, based on traditional Chinese medicine, initially led to skepticism within modern medicine. In the last century, Jisheng Han et al. clarified the spatial and temporal events and related mechanisms of acupuncture-induced analgesia. This is a historic milestone of utilizing biomedical technologies to elucidate acupuncture-induced analgesia mechanisms.

Early preclinical studies indicated that acupuncture stimulates Aδ and C afferent nerves and promotes secretion of endogenous opioid peptide, as well as the reduction in activities of norepinephrine and serotonin systems. With the deepening of basic research on clinical patients, the nodes in default mode and frontoparietal networks have been identified.^651^ The connectivity between the amygdala, right middle cingulate cortex and temporal gyrus is enhanced in patients with nociplastic pain.^652^ Activities of the thalamus, caudate, claustrum and lentiform are likewise modulated.^653^ Importantly, there are typical differences between acupuncture and sham control groups, allaying the concerns of the placebo effect.^652^ Molecular mechanisms underlying acupuncture are considerably diverse, including epigenetic modification, PTMs, non-coding RNAs, inflammasome and microbiota.^41,204,574,654,655^ However, most studies merely reveal their changes following acupuncture, which may be accompanying effects, rather than action mechanisms. The causal studies using rescuing experiments are needed. Taken together, acupuncture is a promising therapy for pain management, but requires further exploration to develop individualized regimens.

### Neuromodulation and brain–computer interface (BCI)

Neuromodulation refers to the approaches that directly or indirectly implant electrodes into innervation regions to improve pathological changes and clinical symptoms. Brain, spinal cord, vagus, sacral nerve, auditory nerve, etc. are all the interventional targets of invasive neuromodulation. Deep brain stimulation has garnered the most attention with the deepening understanding in brain region functions. The sensory thalamus, PAG, ACC and periventricular gray matter are main anatomic regions of deep brain stimulation.^656^ In contrast, transcranial alternating current stimulation represents noninvasive neuromodulation. Though less potent, its safety and convenience may propel it to the forefront of future research. In summary, neuromodulation has proven effective in clinical trials for chronic pain management.^657,658^

Spinal cord stimulation refers to a pain management technique that involves implanting electrodes into the epidural space in the spinal cord for modulating neural electrophysiology. The non-nociceptive electrical signals can inhibit the transmission of nociceptive signals by stimulating the large diameter Aβ-fibers. The most common indications of spinal cord stimulation are complex regional pain syndrome, failed back surgery syndrome and peripheral neuropathy induced by ischemia, herpes zoster or diabetes. Many clinical trials and meta-analysis have verified the efficacy of spinal cord stimulation as a supplementary approach in attenuating neuropathic pain, especially for complex regional pain syndrome and failed back surgery syndrome.^659,660^ It has also been recommended as a third-line therapy for patients who have failed to respond to gabapentinoids and antidepressants.^661^ In addition to the tonic stimulation, some novel stimulation waveforms have been proposed, including burst, high frequency and close-loop stimulation. These further enhance pain relief or reduce the risks associated with paresthesia perception.^662^

Although spinal cord stimulation was initiated based on Gate control theory, the extensive regulatory mechanisms have been gradually uncovered by many preclinical studies. Opioid system is involved in the effects of spinal cord stimulation and the stimulation at different frequencies rely on different endorphins and opioid receptors.^663^ The expression of CB receptors is upregulated following spinal cord stimulation treatment. Nociceptive-evoked activation of supraspinal areas, such as the locus coeruleus, RVM, reticular formation and PAG, can be inhibited by spinal cord stimulation.^664,665^ The descending inhibitory system is activated, leading to the release of 5-HT and attenuation of chronic neuropathic pain.^666^ The above mechanisms collectively contribute to the analgesic effects of spinal cord stimulation.

Despite significant achievements in spinal cord stimulation, there are difficulties in positioning specific pain regions, such as low back, knee and groin. The complex anatomy of spinal cord, shunting of electrical stimulation through cerebrospinal fluid and relative displacement of spinal cord in the canalis spinalis all impair the application of spinal cord stimulation.^667^ DRG stimulation can overcome these shortcomings. It simultaneously activates Aβ, Aδ, and C fibers. The cerebrospinal fluid around DRG forms a groove, attenuating the dispersion of electrical currents and avoiding the side effects of paresthesia within peripheral regions. Moreover, it can produce stable currents in the regions that spinal cord stimulation hardly achieves. According to the existing evidences from clinical trials, DRG stimulation has been selected as the primary treatment of lower limb type I or II complex regional pain syndrome.^668,669^ Patients suffering from chronic postsurgical inguinal pain, knee pain and types of chronic intractable pain can gain typical benefits from DRG stimulation.^670–672^ Some studies have found that stimulation frequencies are a determinant factor of DRG stimulation efficacy and 20 Hz might become the best choice.^673,674^ There are limitations in clinical investigations into either spinal cord stimulation or DRG stimulation. Although the effectiveness of electrical stimulation has been proved, the concrete regimens, such as stimulation frequencies, treatment interval, best indications, need further exploration. Their surgical characteristics make it difficult to set standard sham groups and adhere to blinding principles, affecting the reliability of current clinical data. More high-quality real-world studies should be conducted to compensate for these shortcomings.

The innovation of BCI has ushered neuromodulation into a new era. Preclinical and clinical research has shown its potential in alleviating neuropathic pain. Hence, in this section, we highlight this cutting-edge technique. Pain perception drives fluctuations in the brain network. Extracting features of this process may provide the sources of decoding pain perception with great accuracy.^675^ BCI can analyze the data supported by AI and produce real-time neuromodulation on multiple regions to mitigate pain. The S1, ACC, and PFC are the crucial targets of BCI. More importantly, the regulatory effects are not unilateral. Brain has been found to actively communicate with BCI and change its responses to pain, embodied by enhanced activities of the ACC and PAG and modulation of pain attention.^676^

The studies aiming at prompting more extensive use are ongoing. Patients with phantom limb pain have been trained with BCI to control a phantom hand.^677,678^ A three-day training session can alleviate pain perception for more than 1 week.^679^ In contrast, another study indicated that despite the enhanced discriminability for movement and prosthetic control, overconcentration on the phantom hand driven by BCI intensifies neuropathic pain. Dissociation between prosthetic and phantom hands is a more feasible way for analgesia.^680^ A multisensory intervention strategy consisting of BCI, virtual reality, and transcutaneous electrical nerve stimulation sharply increases the efficiency of decoding pain memory and attenuating neuropathic pain.^681^ The invention of a home-use, patient-managed BCI device has further accelerated the translation of BCI.^682^ More importantly, another application of BCI is assisting movement for paralytic patients. Pain perception has been found to damage the performance of BCI on motor system control.^683^ These findings underscore the broader significance of these advancements, extending beyond mere pain relief.

### Microbial intervention

The breadth of research on microbiota and pain has guided the development of related approaches to analgesics. Investigations into associations between microbiota dysbiosis and pain progression have promoted the translation of novel interventional regimens, mainly including probiotics supplementation and fecal microbiota transplantation (FMT). Notably, given the natural associations between microbiota and gastrointestinal tract, most studies focus on therapies against abdominal pain, particularly IBS.

Probiotic supplementation has exhibited translational value. In the preclinical research, administration of *Saccharomyces boulardii* reduces colonic TRPV1 expression and alleviates pain sensation in an IBS model.^684^ *Lactobacillus paracasei* and butyrate-producing *Roseburia hominis* can respectively attenuate visceral hypersensitivity through mitigating dysfunctions of gut homeostasis.^685,686^ *Bifidobacterium dentium* and *Lactococcus lactis* both have properties of enzymatic decarboxylation of glutamate. Their analgesic effectiveness by GABA production has been detected in visceral hypersensitivity models.^397,687^ The nociceptive perception induced by 5-HT is ameliorated by *Lactobacillus plantarum* through downregulating responses of the HPA axis.^688^ The efficacy of probiotic supplementation have also been confirmed by clinical trials.^689,690^ In addition to natural probiotics, researchers have attempted to exploit the advantages of bioengineering. Genetically engineered *Lactococcus lactis* delivering in-situ IL-10 and IL-22 have been successfully generated. They effectively alleviate visceral hypersensitivity.^691,692^

Probiotics have promising potentials in treating other diseases. For example, the achievements of probiotic application in migraine therapy are relatively mature. Multispecies probiotic strategies have stepped into the validation phase of clinical trials and have exhibited desirable therapeutic effects.^693^ The formation of dental plaque biofilms is recognized as important contributors of oral diseases. A cocktail comprised of 5 bacteriophages targeting *Salmonella typhimurium* can rapidly inhibit biofilms produced by this pathogenic bacterium on the surfaces of teeth, alleviating caries-induced pain in vitro.^694^ In the model of chemotherapy-induced neuropathic pain, elimination of irinotecan-elicited microbiota communities using antibiotic administration effectively relieves hyperalgesia through inactivating TLR4-dependent mechanisms.^403^

Probiotic supplementation mainly influences specific bacterial species and hardly cause global effects on microbiome, which can be compensated by the whole microbiome transplants. In fact, oral consumption of fecal juice was employed to treat patients with food poisoning or diarrhea 1700 years ago. FMT from healthy controls effectively alleviates visceral hypersensitivity both in preclinical and clinical research.^695,696^ Repeated FMT treatment can clinically compensate for the unsustainable curative effects of a single intervention.^697^ The effectiveness and safety of FMT via different delivery routes have also been confirmed in clinical patients, including nasal feeding, enteroclysis and oral administration^697–699^. The success rates of FMT are dependent on microbial diversity, stability and allocation between donors and recipients.^696,697^ Regretfully, sufficient evidence for establishing rigorous criteria for FMT matching, similar to those for clinical transfusion and organ transplantation, is lacking and eagerly required.

### Gene therapy

The burgeoning advancements in bioengineering have significantly augmented the application of gene editing in both basic and clinical research. The identification of pivotal regulators lays a robust theoretical groundwork for gene therapies. Gene therapy methodologies can be categorized into two types based on delivery systems: viral infection and naked plasmids. Viral infection exploits the innate ability of viruses to transport exogenous genetic material into host cells. Viruses are engineered to carry gene editing systems, mainly including zinc-finger nucleases, transcription activator-like effector nucleases and the Clustered Regularly Interspaced Short Palindromic Repeats (CRISPR) system. Undoubtedly, CRISPR/Cas9 technique has garnered considerable attention in current studies. Its recent endorsement for clinical application further underscores its substantial potential. Naked plasmids offer a viable alternative to circumvent the limitations of viral infection. The transfection of plasmids, encoding specific target genes, into cells, can modulate expression of particular RNAs or proteins, thereby influencing pain perception.

A single-arm clinical trial constructed a delivery system using herpes simplex virus to increase preproenkephalin levels in DRG. This methodology efficaciously mitigated pain perception in patients with intractable focal cancer pain.^23^ This study inspires follow-up basic and clinical investigations into viral delivery systems. Some preclinical studies further expanded the potential of gene therapy. Ion channels are important targets in gene therapy. A study focusing on the genetic variations of TRPV1 conducted the CRISPR/Cas9-mediated introduction of a K710N TRPV1 missense variant, resulting in reduced calcium influx and dampened neuronal excitability. It decreased calcium influx and inhibited neuronal excitability, suppressing nociceptive and neuropathic pain.^700^ CBD3 is a peptide aptamer that antagonizes activity of Cav2.2, and delivery of CBD3-encoding gene via adeno-associated virus (AAV) alleviates neuropathic pain.^701^ The integration of glutamate-gated chloride channel silences sensory neurons without affecting motor or proprioceptive functions, attesting to the precision and safety of AAV vectors.^702^

Intriguingly, basic studies have leveraged epigenetic and PTM mechanisms to modulate Nav1.7 expression. The CRISPR-dCas9 system guides inactivated Cas9 protein to *Scn9a* and circumvolutes the target gene instead of editing the sequences, thereby epigenetically blocking *Scn9a* transcription. It exerts long-lasting analgesia for inflammatory and neuropathy-induced pain.^69^ Upregulation of SENP1 via CRISPR system reduces Nav1.7 expression through regulating CRMP2 deSUMOylation.^303^ Remolding phosphorylation sites on histone H3.1 by AAV raises the threshold for thermal nociception.^703^ The indirect regulatory functions of gene therapies may offer more rapid responses and enhanced safety based on the features of epigenetics and PTMs. Furthermore, introduction of the ASC-encoding gene effectively suppresses the schwannoma growth and mitigates associated cancer pain perception.^704,705^ A combinational gene delivery strategy, aiming for simultaneous modulation of neuronal excitability and neuroinflammation, has shown promising results.^706^

Naked plasmids encoding hepatocyte growth factor (HGF) represent a potent approach for treating ischemic diseases. Their role in pain modulation has recently come to light. Topical administration of the naked HGF plasmids suppresses macrophage infiltration in DRG, concomitantly diminishing the release of proinflammatory factors, including IL-1β, IL-6, and TNF-α.^707,708^ This pain relief strategy has passed phase III study on diabetic peripheral neuropathy,^709^ which is the gene therapy closet to clinical application to date. Similarly, IL-10 delivered by naked plasmids induces lasting neuropathic pain suppression independently of its endogenous form.^710^

Taken together, the dual-track development of gene therapies has opened a new direction for upcoming studies. Some future prospects need to be highlighted. Although clinical trials have proven the short-term safety of gene therapies, long-term follow-up is essential to identify underlying risks, given the typical irreversibility of gene editing. Moreover, beyond Cas9, additional genetic scissors have been identified, offering superior applicability.^711^ Loading with the new systems may further improve the efficacy of pain management. Lastly, while current studies focus on hyperalgesia, the phenomenon of pain loss remains under-investigated. Gene mutation is a critical cause of pain loss. Therefore, it is speculated that gene therapies could be effective in this domain.

### Monoclonal antibody

Monoclonal antibody, akin to gene therapy, are part of novel strategies of targeted treatment. Monoclonal antibody inhibits functions of target proteins, which is widely applied in treating cancer and autoimmune diseases. In the pain research, CGRP monoclonal antibodies are undoubtedly the drugs with remarkable performances. Erenumab, fremanezumab, galcanezumab and eptinezumab, targeting CGRP or its receptors, have been approved by FDA to prevent migraine flares with applicability to a broad population. Current trials have been further expanding indications like post-traumatic headache and trigeminal neuralgia^712,713^ and exploring administration routes like oral and intranasal administration.^714,715^

Antibodies targeting NGF have also been proved effective in relieving pain associated with various diseases based on clinical and preclinical research. A meta-analysis has shown that NGF antibodies effectively alleviate symptoms of hip and knee osteoarthritis. Whereas, the drugs may increase risks in therapy discontinuation due to side effects.^716^ Basic studies have exhibited their additional potential in treating postoperative and diabetic neuropathy pain.^717,718^ Furthermore, Antibodies targeting proinflammatory factors, like TNF-α and IL-6, are clinically used for immune disorders, naturally attenuating concomitant pain. Neuropathic pain can also be mitigated by these antibodies,^719^ suggesting there may be overlaps in mechanisms of inflammation and neuropathic pain. Other monoclonal antibodies being investigated are the candidates for clinical pain management, like antibodies against functions of HMGB1, TrkA and PAR2.^720–722^ Modern bioengineering technologies spawn antibodies targeting more direct molecules, including Nav1.7 and TRPV1.^723,724^ The novel monoclonal antibodies proved by preclinical research are listed in Table 3. Despite insufficient evidence on their effectiveness and safety, development of new targets and mechanism-based approaches is an inexorable trend.

**Table 3:** The preclinical research progress in novel monoclonal antibodies for pain relief

Target	Antibody	Disease	Experimental model	Effect	Ref.
Nav1.7	SVmab	Paclitaxel-induced neuropathic pain	In-vitro HEK293 and human DRG neurons	SVmab blocks Na^+^ currents and neuronal excitability.	^723^
P2X3	12D4	Inflammation and visceral hypersensitivity	HEK-293 cell line and primary DRG neurons in vitro and female rats treated with formalin, CFA and TNBS in vivo	12D4 binds to inactivated state of P2X3 and significantly mitigates visceral pain.	^764^
P2X4R	scFv95	Trigeminal neuralgia	Constriction of the trigeminal infraorbital nerve in male and female BALB/c mice	scFv95 completely reverses the neuropathic pain only in the males.	^765^
BDNF	R3bH01	Peripheral nerve injury	SNI and tibial nerve transection in male SD rats	R3bH01 inhibits hyperactivity to heat and mechanical stimuli of sensory neurons in a dose-dependent manner.	^766^
HMGB1	anti-HMGB1 nAb	Trigeminal neuralgia	Constriction of distal infraorbital nerve in male ddY mice	Anti-HMGB1 nAb mitigates neuropathic pain through blocking activation of immune cells.	^720^
TrkA	MNAC13	Inflammation and nerve injury	Treatment with formalin and CCI in male CD1 mice	MNAC13 alleviates hyperalgesia and has synergistic effects in combination with opioids.	^721^
PAR-2	PAR650097	Migraine	Injection of CGRP or SLIGRL in female C57BL/6 J mice	PAR650097 prevents pain perception of both CGRP-dependent and independent migraine.	^722^
PD-L1	Nivolumab	Bone cancer pain	Inoculation of lung cancer cells into male and female C57BL/6 J mice	Nivolumab suppresses bone cancer pain by suppressing osteoclastogenesis.	^767^
RAGE	11E6	Inflammation and nerve injury	CFA stimulation or CCI in male C57BL/6 J mice	11E6 decreases mechanical hypersensitivity in a dose-dependent manner.	^768^
CD11D	anti-CD11d mAb	Spinal cord injury	The laminectomy at T11 in male Wistar rats	No significant efficacies of anti-CD11d mAb are observed.	^769^
GlyRα3	FAbs 9A11, 19C8 and 14E3	-	HEK293 cell line and male SD rats	Selective inhibition of GlyRα3 may have potentials in treating hyperalgesia and has good safety in vivo.	^561^
MMP9	MMP9 mAb	Paclitaxel-induced pain	Administration of paclitaxel in male and female CD1 mice	MMP9 mAb alleviates paclitaxel-induced pain through inhibiting inflammatory responses to oxidative stress and loss of intraepidermal nerve fibers.	^770^
TRPM8	ACC-049 (polyclonal antibody)	-	Primary DRG neurons derived from male SD rats	The effects of ACC-049 on TRPM8 blockade are verified.	^771^
VEGFR-1	D16F7	Chemotherapy-induced pain (oxaliplatin, paclitaxel and vincristine)	Administration of chemotherapeutic drugs in male CD1 mice	D16F7 inhibits the development of chemotherapy-induced pain through interfering with astrocyte-neuron crosstalk.	^772^
RGMa	AE12-1 and AE12-1Y	Spinal cord injury	Extradural impact-compression in female Wistar rats	AE12-1 and AE12-1Y enhance neuronal survival capability, plasticity of descending serotonergic pathways and axonal regeneration. The activation of microglia and CGRP expression are also inhibited, alleviating pain perception.	^773^
OxPL	E06	Inflammation	CFA treatment in male Wistar rats and female Lewis rats	E06 suppresses hyperalgesia and CGRP release by blocking TRPV1 and TRPA1.	^774^

*CCI* chronic constriction injury, *CFA* complete Freund’s adjuvant, *DRG* dorsal root ganglion, *SD* Sprague-Dawley, *TNBS* trinitro-benzene-sulfonic acid

### Stem cell therapy

Stem cell therapy is an important branch of regenerative medicine. It has been extensively applied in treating various diseases. It is categorized into placenta and umbilical cord-, hematopoietic-, bone marrow- and adipose-derived types according to the origins. Their general mechanisms include the following three aspects. The direct way is rapidly dividing at lesions and preparing injured tissues. Stem cells also release a batch of trophic factors, like TGF-β, vascular endothelial growth factor (VEGF) and other cytokines. The paracrine function is regarded as more important than direct engraftment for chronic diseases, because the persistent efficacy can be observed after displacement and absorbance of stem cells.^725^ Stem cells are also able to exert global immunomodulation, such as inhibition of monocyte differentiation to dendritic cells, T cell development and NK cell regulation.

In the preclinical research, stem cells derived from bone marrow, adipose and peripheral nerve significantly attenuate neuropathic pain.^726–728^ The opioid tolerance is reversed by stem cell transplantation.^729^ VEGF is a potent regulator of counteracting complex neuropathy. Bioengineered stem cells expressing VEGF exhibit better efficacy in pain relief of patients with Parkinson’s disease.^730^ Another notable finding is the significance of stem cell spheroids, which outperform the monolayer-cultured stem cells. The capability of neurotrophic factor secretion is dramatically enhanced, accompanied with impaired immune cell infiltration. The in-vivo survival time of spheroids is also extended.^731,732^ Development of stem cell spheroid treatment may be a promising direction. The effectiveness and short-term safety of stem cell therapy in pain induced by neuromuscular skeletal disorders and neuropathy have been validated by clinical trials.^733,734^ However, the long-term risks in carcinogenesis and autoimmune disease induction, need careful evaluation.

In conclusion, various therapeutic strategies have been developed and applied in clinical pain management. Besides the abovementioned modalities, there are other developing approaches, such as immunotherapy, nerve regeneration, diet management and surgical intervention. Another trend is integration with biomaterials. They are able to potentiate targeting abilities of loaded pharmaceuticals, consequently enhancing potencies for pain relief and avoiding undesirable side effects.^735^ However, clinical requirement for pain relief is far from satisfaction, again implying the complexity of pain sensation. Multidisciplinary cooperation, supported by high-quality preclinical and clinical research, may be a key solution to this dilemma.

## Shortcomings of the current pain research

The strength of evidence in existing research, particularly regarding brain regions, is generally weak. Studies often detected activity alterations in brain regions after pain induction or intervention, along with synergistic regional reactions. On the basis of these data, they inferred that some brain regions and functional connectivity might participate in pain modulation. Similar situations were also observed in the research on specific inflammatory mediators, neurotransmitters and molecular regulators. These findings are useful for developing novel diagnostic biomarkers and screening out potential targets. Nevertheless, they fall short in supporting concrete conclusions about pain etiology and treatment. Researchers should exercise caution in drawing conclusions based on assumptions from previous reports and established knowledge. Such weak evidence may obscure the true nature of pain modulation. For instance, the accepted view that TRPV1 activation serves as a marker of hypersensitivity, used in pain perception assessment. However, CB1-dependent TRPV1 overactivation is an important mechanism in dipyrone-induced analgesia,^572^ overturning the stereotype regarding TRPV1. β-arrestin 2 has been recognized as a critical mediator of opioid-induced respiratory depression based on previous studies. Its role is now questioned, as β-arrestin 2 knockout does not affect respiratory rhythms.^606^ Otherwise, GABA is a negative regulator of hyperalgesia, but its depression comorbidity-inducing effects^473^ are often overlooked. Therefore, the lack of studies related to causal and mechanistic data increases difficulties in identifying more valuable targets.

The experimental methods require significant improvement. The reliance on mouse and rat models for human disease studies has been long debated due to substantial differences in nervous systems.^736^ This species gap may introduce biases in clinical translation. Additionally, gene editing and controlling certain substance levels in in-vivo models are relatively challenging compared to in-vitro models, slowing progress in exploring mechanisms underlying pain modulation. As mentioned previously, pain-related disorders are multifaceted. A single modeling approach cannot fully simulate human pain sensation, further affecting the credibility of current research. Fortunately, scientists are addressing this by developing composite modeling strategies. Moreover, commonly used pain indicators, such as c-fos expression, ion channel activity and hormone levels, may not accurately reflect pain severity in experimental animals. The representativeness of these indicators warrants scrutiny and validation. The above problems, of course, are universal flaws of basic experiments and are not unique to pain research.

The potential of multi-omics and high-resolution approaches remain underexploited. The authors only find that microbiome combined with metabolomics is widely used in the existing studies on microbiota and pain. While the findings have deepened our understanding and advanced pain diagnosis and treatment, it risks forming research stereotypes of the multi-omics pattern of microbiome plus metabolomics. Furthermore, several recent studies analyzed basic structures and mechanisms underlying responses to nociception using single-cell transcriptome. However, other omics approaches, like transcriptomes, proteomics and spatial omics, are overlooked, especially the combinational application. The multi-omics approaches and high-resolution have led to significant discoveries in other nervous system diseases, such as Alzheimer’s disease, depression and autism spectrum disorder.^737–739^ By contrast, few pioneer investigations have preliminarily shown the potential of transcriptomes and proteomics in exploring pain modulation,^740,741^ implying that pain research lags due to underutilization of multi-omics analysis. On the other hand, the efficiencies of omics data analysis integration are relatively low. The depth of omics data analysis is limited, especially for the basic research for investigating pain causality. In summary, the small data pool and rough analytical tools of omics both hamper the clinical progress in pain relief.

Data on pain modulation mechanisms and therapeutic regimens often present contradictions. The prime example is that the performances of specific therapeutic approaches are different across clinical trials, particularly in alternative and complementary therapies for pain management. Such discrepancies, which are influenced by numerous unpredictable confounding factors, are common in clinical research. However, similar inconsistencies are also observed in preclinical research. For instance, 17β-estradiol is reported as both a promoter of pain in females and a protective agent against hyperalgesia.^742^ Moreover, the changes in expression of HDACs in response to pain modeling are controversial.^213,743^ These opposing conclusions without reasonable explanations create barriers to deeper studies. Thus, monism is unsuitable for estimating the roles of pain regulators, which may function distinctly under various conditions.

The progress in clinical trials for pain management is slow. The scarcity of large-sample, multicenter clinical trials hamper clinical translation of novel pain management approaches. Limited patient inclusion reduces the practicality of subgroup analysis in identifying potential beneficiaries. Long-shot clinical investigations for high-level evidence-based support are lacking. Moreover, potential bias of publication, favoring studies with positive results, is a serious yet neglected problem. The clinical trials that display negative data or vigilance about the side effects have more difficulties in gaining extensive attention. Although this phenomenon has been improved to some extent recently, the follow-up impact following the previous phenomenon will persistently exist, probably causing more waste of basic and clinical resources.

Some mechanisms remain underexplored, especially in burgeoning research areas. For instance, current studies primarily focus on a few PTMs, including phosphorylation, ubiquitination, SUMOylation and glycosylation. This does not mean the unimportant roles of other PTMs. Instead, sporadic studies have reported their potentials in regulating nociceptor sensitivity and molecular activity, implying the unidentified PTM networks in pain modulation. Similarly, NLRP3 inflammasome is undisputedly a key mediator in hyperalgesia. This research trend may make other inflammasomes with properties in pain modulation, like NLRP2, ignorable. Otherwise, in the research on microbiota and pain, nonbacterial microbiota, like fungi, which regulate pain sensation, receive minimal attention. Basic knowledge about pain and biofilms, an important accessory structure of microbial community, is also limited. The sluggish paces with research frontiers may result in missing out on many diagnostic and therapeutic methods.

## Future perspectives of the pain research

Improvement in human-based in-vitro systems as research models. To surpass the inherent limitations of experimental animals as in-vivo models and in-vitro two-dimensional cells models (cell lines and induced pluripotent stem cells), the utilization of organoid and organs-on-a-chip technologies is essential in the follow-up studies. Organoids are the three-dimensional culture systems derived from self-organizing stem cells. Organs-on-a-chip systems are in-vitro microfluidic devices containing the cell types of interest in close recapitulation of the original tissue structure, function, and physiology. The advent of these two in-vitro culture biotechnologies provides more opportunities for narrowing the gap in mechanistic insights into pain. Organoids and organs-on-a-chip systems possess both the maneuverability of in-vitro cell line models and integrity of in-vivo animal models. They have been widely adopted in various research areas, establishing a robust foundation for data production. Nevertheless, few studies on pain perception chose them as experimental models, likely due to technological and financial barriers. The application of new biotechnologies always requires powerful supports of experimental technique and funding. To this end, just as developing trends of other biotechnologies, like high-throughput sequencing and antibody preparation, extensive commercialization of organoids and organs-on-a-chip systems may sharply reduce their application thresholds, fully unlocking research potential. Moreover, improvement in extracellular matrix structures and functions is important likewise. Three-dimensional bioprinting, microfluidics device and biomaterials should be further developed to generate organs with fine and complex structures incorporated with vasculature and innervation networks. Furthermore, cryoelectron microscopy (cryo-EM) is an emerging technique for analyzing molecular structures, providing in-depth evidences for investigating mechanisms and exploring new analgesic drugs. We are glad to see that cryo-EM has been extensively used in pain research (Table 4). More studies are required to illustrate functions of critical regulators in pain modulation.

**Table 4:** The research progress in structure analysis of pain-associated molecules using cryo-EM

Molecule	Aim	Significance	Ref.
NaV1.3	To investigate the mechanisms by which bulleyaconitine and a selective inhibitor ICA121431 block Nav1.3.	Bulleyaconitine resides around domain I-II fenestration and blocks ion channels. ICA121431 stabilizes the activation state of Nav1.3 through preferentially binding to the domain IV voltage-sensor.	^775^
Nav1.7	To investigate the structures of Nav1.7 bound with riluzole and lamotrigine.	Riluzole binds to the central cavity of Nav1.7 pore, whereas lamotrigine has dual-pocket mechanisms. In addition to the above site, lamotrigine additionally resides in the beneath intracellular gate, synergistically inhibiting Nav1.7 activity.	^776^
Nav1.7	To discriminate the structural basis of various Nav1.7 antagonists.	Carbamazepine, bupivacaine and lacosamide reside in a binding site beneath the intracellular gate. Lacosamide closely binds to the selectivity filter. Vinpocetine plugs in the III-IV fenestrations, whereas vixotrigine can penetrate the IV-I fenestrations.	^777^
Nav1.7	To investigate the structural basis of Nav1.7 mutants.	The first voltage-sensing domain exhibits inactivation conformation, which is attributable to transference of three gating charge residues into the cytosolic side, leading to intracellular gate rearrangement and contraction. The data provide proofs for understanding pain-related mutations.	^778^
Cav1.2	To identify Cav1.2 structures and the structural mechanisms of the calcium channel blockers.	Pinaverium bromide, an antispasmodic drug, is inserted into IFM-binding site and induces global alterations of Cav1.2, while calciseptine binds to the shoulder of the pore domain.	^779^
Cav2.2	To elucidate the inactivation state of Cav2.2.	S6 helices in the second voltage-sensing domain are essential for maintaining inactivation, and a W-helix from the domain II–III linker further stabilizes the close state of Cav2.2. This finding may contribute to the development of novel Cav2.2 blockers.	^780^
Cav2.3	To explore the structures of Cav2.3 in complex with α2δ-1 and β3 subunits, together with the differences with a Cav2.3 mutant.	Cav2.3 has the high similarity in cellular structures with Cav2.2. CH2II helix is deleted in Cav2.3 mutant, which is critical for stabilizing the inactivated conformation.	^781^
Cav3.1	To show the structures of Cav3.1 alone and in complex with Z944, a selective blocker of Cav3.1.	The structural mechanisms of Z944 residing in the central cavity of the pore domain of Cav3.1 are revealed, deepening the understanding in distinct channel properties of VGCCs.	^782^
KCNQ4	To explore the structures of KCNQ4 and in complex with an opener retigabine or an inhibitor linopirdine.	Signaling factor PIP2 is inserted into the cavity of voltage-sensing domain for channel activity modulation. The fenestration and cytosolic cavity are the binding regions of retigabine and linopirdine, respectively. The data lay foundations for developing new drugs targeting KCNQ4.	^783^
TRPV3	To display the structure of TRPV3-dyclonine complex.	Dyclonine sticks into the channel pore and blocks the connection with membrane environments.	^784^
TRPV3	To identify the opening structure of TRPV3.	The structural changes in both the outer pore and the intracellular gate occur in channel opening. S6 helices in the gate are further elongated and rotated.	^785^
CB1, CB2	To identify the structures of CB1 and CB2 in complex with Gi.	The structures of Gi complexes with CB1 or CB2 are obtained, which provide proofs for clarifying their selectivity. Cholesterol is identified as an endogenous allosteric modulator of CB1.	^786^
CB2	To investigate the mechanisms underlying activation and signaling of CB2.	The structures of CB2-G_i_ complex and interactions with potent agonist WIN 55,212-2 are identified. Moreover, a unique motif in CB2 is found to underlie its specificity of G_i_ coupling.	^787^
GABA_A_	To explore the structural basis of α1β3γ2L, a major isoform of GABA_A_ receptor.	Intracellular M3-M4 loops in α1β3γ2L are vulnerable to physiological and pharmacological regulation, which is an important reason of heteromeric organization of the GABA_A_ receptor.	^788^
GABA_A_	To explore the principles of pentamer formation of GABA_A_ receptor.	The permutational landscape of GABA_A_ receptor is depicted based on the theory of differential assembly. GABA_A_ arrangements are found to meanwhile respond to GABA and histamine.	^789^
GABA_B_	To screen the ligands pre-associating with the GABA_B_ receptor.	Transmembrane domains are the key sites of ligands binding to the GABA_B_ receptor. A heterodimer interface between helices 3 and 5 is found to be essential for GABA_B_ inactivation.	^790^
GlyR	To clarify the mechanisms by which THC, the principal psychoactive component in cannabis sativa, modulates GlyR activity.	THC activates GlyR through directly regulating the opening states of the channel pore.	^791^
mGlu2	To elucidate the structures of mGlu2 homodimers and complexes with heterotrimeric G_i_.	An asymmetric TM6-TM6 interface is critical for the extensive rearrangement of transmembrane domains. The mechanisms of family A and B GPCRs rely on the TM6-TM6 interface, while their family C G protein coupling functions in a TM6-independent manner.	^792^
MRGPRX1	To explore the mechanisms underlying the activation of MRGPRX1, a molecule mediating pain perception.	The three structures of MRGPRX1 in complex with opioid peptide fragment BAM8-22, BAM8-22 plus positive allosteric modulator ML382 and a synthetic agonist compound-16 are revealed. The data provide evidence for developing novel non-opioid drugs.	^793^
A_1_R	To clarify the mechanisms underlying MIPS521, a positive allosteric modulator of A1R.	A_1_R binds to a heterotrimer of adenosine, MIPS521 and G_i2_ via an extrahelical lipid-detergent-facing allosteric binding pocket involving helixes 1, 6 and 7. The data provide new evidence for developing allosteric drugs.	^794^
P2X4	To identify the structures of P2X4 in complex with specific antagonists BX430 and BAY-1797.	BX430 and BAY-1797 can both bind to the allosteric site at the subunit interface of the P2X4 extracellular domain.	^795^

*cryo-EM* cryoelectron microscopy, *THC* Δ9-tetrahydrocannabinol

Comprehensive use of omics profiling. Single-cell and spatial omics technologies have become landmark achievements in the technological revolution. Pain research has begun employing single-cell and spatial omics to unravel new mechanisms, such as compiling DRG atlas, identifying mechanistic networks and discovering new cell subtypes.^744^ More focus is needed in areas like analgesic therapy responses, opioid tolerance and pain sensation variations among populations. Furthermore, the integration of multi-omics detection with these high-resolution technologies is absent but greatly needed. The joint analysis of microbiome and metabolomics has already laid a foundation. Expanding omics types and data volumes should be further encouraged. The rapid advancement of AI offers robust data analysis capabilities for multi-omics technologies.^745^ Future studies should try to widen the scope of multi-omics profiling and deepen omics data mining. Recently, the most comprehensive human brain cell map has been disclosed. This work set a benchmark for future omics studies. Although introduction of new omics approaches may disclose more unexplored zones and put more demand on researchers, it will tremendously broaden the horizons concerning pain modulation. The profits in better understanding mechanisms and identifying more related targets can boost the development of pain diagnosis and treatment.

Development of noninvasive methods for pain diagnosis. Due to the subjectivity and heterogeneity of pain perception, clinical pain evaluation typically depends on scales and assessment by doctors, which are not always reliable. Novel approaches like gene biomarkers and brain imaging have yet to effectively address these limitations. Based on the fact, the future directions can be concluded into four aspects. i) Collection of more high-level evidence-based medical data. Since existing data have shown their optimal performance, trials with larger samples and subsequent commercialization processes should be advocated. This measure may allow patients to benefit from these achievements at an earlier time. The differences of pain perception in various subpopulations can be better excavated. ii) Rapid diagnosis based on molecular targets and easily accessible samples. Current approaches have difficulties in balancing efficiency and accuracy. Novel bioengineering techniques are favorable for overcoming this dilemma. Lin and colleagues set a good example. They develop a nanochip detecting saliva CGRP concentrations for migraine diagnosis. The results can be obtained within 10 min.^746^ More investigations into rapid diagnosis need to be encouraged. iii) Streamlining detected indicators. Some studies used ten more biomarkers or global high-throughput data to train pain models. Despite theoretically good performances, they may not conform to clinical reality due to their high cost and complexity. Herein, the authors call for investigations into applications of smaller-scale indicators. iv) Multimodal diagnostic methods. Although we encourage decreases in included indicators of one diagnostic methods, the advantages of multimodal approaches should be highlighted. Multimodal approaches can mutually compensate for shortcomings of single method, like BCI combined with skin conductance.^681^ The integration of AI in multimodal approaches could offer the most efficient ways to meet realistic conditions.

Extension of novel strategies for pain management. i) Activation of immunoreaction against pain. Immune disorders significantly contribute to hyperalgesia, and most approaches pertain to the passively mitigation. The success of cancer immunotherapy hints that the immune system may be another key to pain relief. For instance, Sara et al. creatively employed microbiota antigens to activate specific immunity, obliterating visceral hypersensitivity-associated microbiota.^747^ This research is opening new possibilities in this field. On the basis of the reported advantages of immunotherapy, responders are likely to obtain more benefits from it, embodied by lasting medical effects, mild side effects and good tolerance.^748^ ii) Leveraging biomaterial superiority. Interdisciplinary investigation is a promising approach to promote translation, mainly including efficacy enhancement, potency prolongation, side effect mitigation and cost minimization. Despite its promise, collaborations involving biomaterials remain scarce. The need for more high-quality studies is evident. iii) Formation of comprehensive treatment strategies. The characteristics of pain, as a multifactorial disease, necessitates multifaceted interventions, including, analgesics, emotional management, social relations improvement, together with alternative and complementary therapies. Research is shifting towards examining the synergistic effects of combined therapies, such as analgesic drugs with CBT and cell stem therapy with probiotics.^749,750^ More research investment should be conducted to make one plus one larger than two. iv) Learning from nature. Many breakthroughs in pain research are inspired by natural phenomena. Certain natural compounds are found to have analgesic efficacy, potentially more effective than synthetic drugs.^751,752^ The natural compounds and endogenous substances, such as melatonin, curcumin and peppermint, with a history of safe use, offer wide regulatory mechanisms. The advantages can effectively decrease risks associated with new drug development.

## Conclusions

**Fig. 10:**
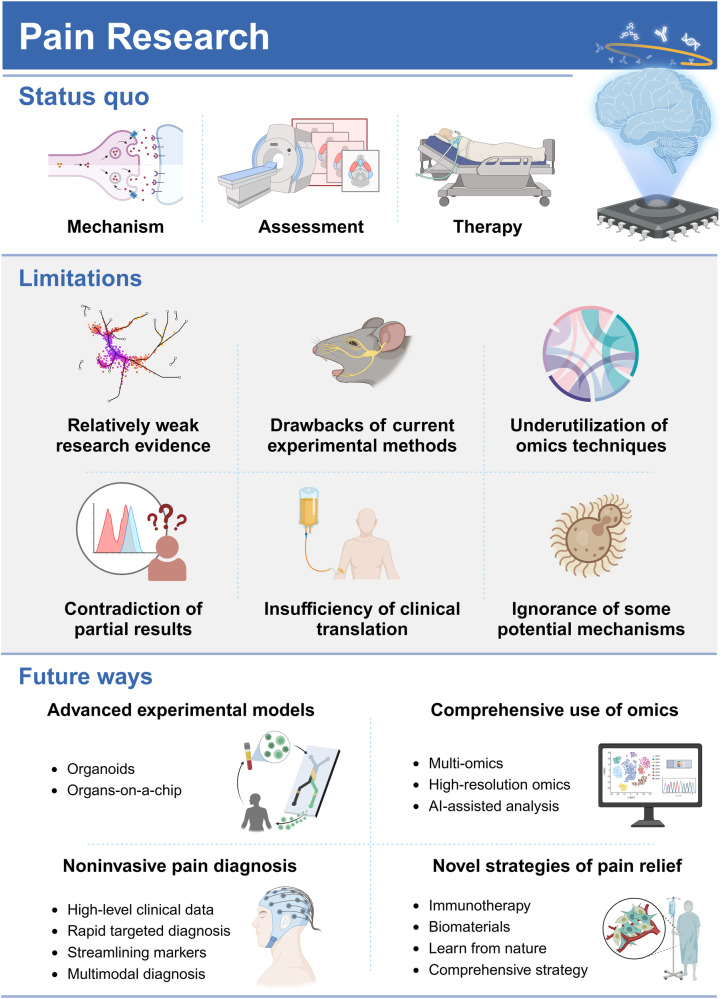
The status quo, limitations and future perspectives of the pain research. Most current preclinical and clinical studies in the pain field focused on its mechanisms, assessment and therapy. However, there are some limitations as follows. i) Current research evidence is relatively weak and the underlying mechanisms remain largely unknown. ii) The in-vitro and in-vivo experimental models cannot thoroughly mimic the clinical conditions of pain. iii) The research value of omics techniques is not fully exploited. iv) There are contradictions in the results from some studies. v) Current progress in clinical translation of pain research achievements is far from clinical requirements. vi) Some potential mechanisms underlying pain modulation should be emphasized, like the regulatory role of fungi. Herein, we propose four future perspectives for pain research, including development of advanced experimental models, comprehensive application of omics, emphasis on noninvasive pain diagnosis and optimization of strategies for pain relief

Pain, a kind of universal experience, brings about overwhelming physical and mental distress. Attenuating pain is a fundamental right of patients. Extensive preclinical and clinical studies have delved into pain pathology and the molecular mechanisms of pain modulation. Key brain regions and critical molecules have been identified involved in pain perception. The research achievements regarding TRPV1, TRPM8 and Piezo even garner recognition of the Nobel prize. The significant progress has facilitated a shift from empirical management to personalized interventions. A range of promising diagnostic and therapeutic approaches have emerged. Some Chinese traditional therapies are rehabilitated by modern medical evidence. Multidisciplinary collaborations have further advanced the field and integrated cutting-edge technologies, such as virtual reality, biomaterials, and high-resolution omics technologies. However, current research limitations are impeding further advancements in pain management. This review suggests several potential directions for future research (Fig. 10). The authors believe that the unpleasure experience of pain will be eradicated as neuroscience continues to evolve.
